# DNA methylation at birth and lateral ventricular volume in childhood: a neuroimaging epigenetics study

**DOI:** 10.1111/jcpp.13866

**Published:** 2023-07-19

**Authors:** Mannan Luo, Esther Walton, Alexander Neumann, Chris H. L. Thio, Janine F. Felix, Marinus H. van IJzendoorn, Irene Pappa, Charlotte A. M. Cecil

**Affiliations:** 1 Department of Psychology, Education and Child Studies, Erasmus University Rotterdam, Rotterdam, The Netherlands; 2 Generation R Study Group, Erasmus MC, University Medical Center Rotterdam, Rotterdam, The Netherlands; 3 Department of Psychology, University of Bath, Bath, UK; 4 Department of Child and Adolescent Psychiatry/Psychology, Erasmus MC, University Medical Center Rotterdam, Rotterdam, The Netherlands; 5 Department of Epidemiology, University Medical Center Groningen, University of Groningen, Groningen, The Netherlands; 6 Department of Pediatrics, Erasmus MC, University Medical Center Rotterdam, Rotterdam, The Netherlands; 7 Research Department of Clinical, Educational and Health Psychology, Faculty of Brain Sciences, UCL, University of London, London, UK; 8 Clinical Child and Family Studies, Vrije Universiteit Amsterdam, Amsterdam, The Netherlands; 9 Department of Epidemiology, Erasmus MC, University Medical Center Rotterdam, Rotterdam, The Netherlands; 10 Molecular Epidemiology, Department of Biomedical Data Sciences, Leiden University Medical Center, Leiden, The Netherlands

**Keywords:** cord blood, methylation profile score, psychotic-like experiences, Generation R, ALSPAC, EWAS.

## Abstract

**Background:**

Lateral ventricular volume (LVV) enlargement has been repeatedly linked to schizophrenia; yet, what biological factors shape LVV during early development remain unclear. DNA methylation (DNAm), an essential process for neurodevelopment that is altered in schizophrenia, is a key molecular system of interest.

**Methods:**

In this study, we conducted the first epigenome-wide association study of neonatal DNAm in cord blood with LVV in childhood (measured using T1-weighted brain scans at 10 years), based on data from a large population-based birth cohort, the Generation R Study (*N* = 840). Employing both probe-level and methylation profile score (MPS) approaches, we further examined whether epigenetic modifications identified at birth in cord blood are: (a) also observed cross-sectionally in childhood using peripheral blood DNAm at age of 10 years (Generation R, *N* = 370) and (b) prospectively associated with LVV measured in young adulthood in an all-male sample from the Avon Longitudinal Study of Parents and Children (ALSPAC, *N* = 114).

**Results:**

At birth, DNAm levels at four CpGs (annotated to potassium channel tetramerization domain containing 3, *KCTD3*; SHH signaling and ciliogenesis regulator, *SDCCAG8*; glutaredoxin, *GLRX*) prospectively associated with childhood LVV after genome-wide correction; these genes have been implicated in brain development and psychiatric traits including schizophrenia. An MPS capturing a broader epigenetic profile of LVV – but not individual top hits – showed significant cross-sectional associations with LVV in childhood in Generation R and prospectively associated with LVV in early adulthood within ALSPAC.

**Conclusions:**

This study finds suggestive evidence that DNAm at birth prospectively associates with LVV at different life stages, albeit with small effect sizes. The prediction of MPS on LVV in a childhood sample and an independent male adult sample further underscores the stability and reproducibility of DNAm as a potential marker for LVV. Future studies with larger samples and comparable time points across development are needed to further elucidate how DNAm associates with this clinically relevant brain structure and risk for neuropsychiatric disorders, and what factors explain the identified DNAm profile of LVV at birth.

## Introduction

Schizophrenia is an umbrella term encompassing a range of severe and highly heterogeneous psychiatric symptoms (Kahn et al., 2015). The heterogeneity of the disorder complicates our understanding of its potential pathophysiology. Intermediate phenotypes with a greater biological resolution, such as brain structures, may help to reduce this heterogeneity and improve our ability to identify underlying molecular pathways (Birnbaum & Weinberger, 2017). Enlargement of the lateral ventricles is one of the most replicated findings in schizophrenia (Kuo & Pogue-Geile, 2019; Olabi et al., 2011; van Erp et al., 2016), making it a particularly promising neural intermediate phenotype.

The lateral ventricles are the largest cavity of cerebral ventricular systems, taking shape during the embryonic development of the primitive neural tube (Orahilly & Muller, 1990). The development of these ventricles is thought to be influenced by both genetic and environmental influences beginning in utero. In particular, lateral ventricular volume (LVV) shows a twin and familial heritability ranging from 32% to 35% in childhood to 75% in adulthood, and an SNP-based heritability of 20% (Kremen et al., 2012; Vojinovic et al., 2018). Moreover, genetic risk for schizophrenia has been found to act jointly with environmental factors, such as birth complications or postnatal exposure to chronic stress, to predict ventricular enlargement in adolescence and young adulthood (Aas et al., 2013; Bolhuis et al., 2019; Cannon et al., 1993). Yet, how these influences come together at a molecular level to shape early LVV and downstream risk for schizophrenia, which typically manifests years later in adolescence and young adulthood, remains unclear.

Epigenetic processes, such as DNA methylation (DNAm), have emerged as a potential molecular system of interest, as they: (a) regulate gene activity in response to environmental influences beginning in utero (e.g. maternal stress; Kotsakis Ruehlmann et al., 2023) and (b) are partly under genetic control (Min et al., 2021). Furthermore, DNAm plays an important role in healthy neurodevelopment (Dall’Aglio et al., 2018), and disruptions in DNAm have been linked to psychiatric disorders, including schizophrenia (Chen et al., 2020; Richetto & Meyer, 2021; Smigielski, Jagannath, Rössler, Walitza, & Grünblatt, 2020). Most studies to date have associated DNAm with schizophrenia using postmortem brain tissues, which cannot inform on developmental processes in living individuals (Lancaster, Morris, & Connelly, 2018). Neuroimaging epigenetics can help to bridge this gap by combining DNAm from peripheral tissues with in vivo brain imaging techniques, such as structural magnetic resonance imaging (MRI; Walton et al., 2023; Wheater et al., 2020). This approach could provide novel insights into epigenetic processes contributing to brain features associated with schizophrenia risk, such as LVV enlargement. While making mechanistic inferences about the role of peripheral DNAm in brain-based phenotypes is challenging (e.g. due to cross-tissue variability in DNAm), research has increasingly demonstrated the potential of peripheral DNAm as a non-causal biological marker for disease prediction, stratification, and diagnosis (Cecil, Neumann, & Walton, 2023; Min et al., 2021).

To our knowledge, no study has yet examined epigenome-wide DNAm patterns associated with LVV. Although previous studies have linked peripheral DNAm to other brain regions in the context of schizophrenia, such as hippocampal volume, these have primarily used cross-sectional designs in clinical samples of adults (Chen et al., 2020; Wheater et al., 2020). As such, it remains unclear whether DNAm patterns prospectively associate with brain features in the general pediatric population, before the manifestation of later psychotic symptoms. To clarify the direction of DNAm–brain associations and minimize potential confounding, such as by the use of medication, prospective studies linking early DNAm with the developing brain are needed. Furthermore, the focus on DNAm measured at a single time point poses challenges, given that DNAm is highly developmentally dynamic (Mulder et al., 2021), and importantly, that associations with brain-based phenotypes can be temporally specific. For example, growing evidence shows that DNAm at birth is more strongly associated with certain neurodevelopmental outcomes, such as ADHD and social communication deficits (Neumann et al., 2020; Rijlaarsdam, Cecil, Relton, & Barker, 2021), compared with DNAm patterns measured concurrently in childhood (i.e. prospective > - cross-sectional associations). Leveraging repeated measures of epigenetic data is thus key to characterizing how DNAm markers may associate with brain phenotypes at different stages of development. However, little research to date has embedded replication attempts, which is important to establish the reliability and generalizability of findings.

In light of these gaps, we used data from two independent population-based cohorts, the Generation R Study and the Avon Longitudinal Study of Parents and Children (ALSPAC), to characterize early epigenetic correlates of LVV as well as to test the generalizability of our findings across different developmental periods. Based on Generation R, we performed the first epigenome-wide association study (EWAS) examining prospective associations between DNAm at birth (cord blood) and LVV in childhood (age 10 years). To verify the relevance of LVV to psychotic outcomes in the general pediatric population, we also examined the prospective association between child LVV and psychotic-like experiences in adolescence. We then utilized both probe-level and methylation profile score (MPS) approaches to evaluate the generalizability of our findings by: (a) testing whether associations are temporally stable when using DNAm measured at 10 years in Generation R (i.e. cross-sectional associations with LVV), and (b) whether DNAm at birth continues to predict LVV in young adulthood (as opposed to childhood in the discovery analyses) using an independent sample of males from the ALSPAC cohort.

## Methods

### Participants

Primary analyses were conducted using data from the Generation R Study, a population-based prospective cohort from early fetal life onward, based in Rotterdam, the Netherlands. The design and sample characteristics of Generation R have been described in detail elsewhere (Kooijman et al., 2016). For a subsample of 1,396 European singletons DNAm data was collected at birth. Of these, 1,382 remained after quality control and harmonization, including 840 children (50.4% female) with available data on neuroimaging measured at a mean (*SD*) age of 10.1 (0.6) years and relevant covariates. In addition, we included participants with information on self-reported psychotic-like experiences (*N*_hallucinations_ = 2,360, *N*_delusions_ = 1,899) at a mean (*SD*) age of 13.6 (0.4) years. In addition, 370 children had available data on DNAm (based on peripheral whole blood) at age of 10 years, in addition to neuroimaging and relevant covariates. A flowchart of sample selection is described in Figure S1.

The generalizability analyses were performed in an independent population-based prospective cohort, the ALSPAC study in the UK (Boyd et al., 2013; Fraser et al., 2013), based on data from 839 European singletons with available cord blood DNAm at birth. Of these, 114 males had neuroimaging measures at a mean (*SD*) age of 19.5 (0.8) years, and 528 participants (40.9% male) completed a semi-structured interview of psychotic-like experiences at a mean (*SD*) age of 24.4 (0.7) years, respectively. Full cohort descriptions are provided in Appendix S1.

### Measures

Full information on measures of DNAm, structural neuroimaging, and psychotic-like experiences within each cohort is provided in Appendix S1.

#### DNA methylation

Briefly, DNAm was measured in cord blood (Generation R and ALSPAC) and peripheral whole blood at age of 10 years (only in Generation R). Samples were further processed with the Illumina Infinium HumanMethylation450 BeadChip (Illumina Inc., San Diego, CA, USA). Initial quality control was performed using the CPACOR workflow (Lehne et al., 2015) in Generation R and the *meffil* package (Min, Hemani, Davey Smith, Relton, & Suderman, 2018) in R in ALSPAC. To minimize cohort effects, we previously combined samples from the two cohorts and then normalized them as a single dataset (Mulder et al., 2020). Functional normalization (10 control probe principal components, slide included as a random effect) was performed with the *meffil* package in R. Normalization took place on the combined Generation R and ALSPAC data for a total of 485,512 CpG sites. More detailed quality control and normalization steps for the combined dataset have been described previously (Mulder et al., 2020). Methylation levels outside of the lower quartile minus 3 × interquartile range or upper quartile plus 3 × interquartile range were identified as outliers and winsorized.

#### Structural MRI

In Generation R, MRI was acquired at age of 9–11 years on a 3-Tesla MRI system (MR750w, General Electric, Milwaukee, WI, USA) using an 8-channel head coil (White et al., 2018). In ALSPAC, MRI was acquired at age of 18–21 on a 3-Tesla General Electric HDx (GE Medical Systems) using an 8-channel head coil (Sharp et al., 2020). It should be noted that neuroimaging data in ALSPAC were available in male participants only, as they were generated as part of a specific project on the effect of pubertal testosterone on brain development. In both cohorts, automatic volumetric segmentations of the structural T1-weighted images were processed using the FreeSurfer package. Total LVV was standardized using *Z*-score transformation.

#### Psychotic-like experiences (PLE)

In Generation R, we used two measures of psychotic-like experiences which were self-reported by children at age of 13–14 years, including hallucinations assessed with two items from the Youth Self-Report (Ivanova et al., 2007), and delusions assessed with six items from the Kiddie Schedule for Affective Disorders and Schizophrenia (Adriaanse, van Domburgh, Zwirs, Doreleijers, & Veling, 2015; Kaufman et al., 1997). Following previous research, the sum scores of hallucinations were categorized into three groups: no symptoms, mild, and moderate-to-severe symptoms (Bolhuis et al., 2018), whereas the sum score of delusions was used as a continuous variable (Adriaanse et al., 2015). In ALSPAC, psychotic-like experiences at age of 24 were identified through the face-to-face, semi-structured psychosis-like symptom interview (PLIKSi; Zammit et al., 2013). Interviews were conducted by trained psychology graduates in assessment clinics. Total scores from the PLIKSi were recoded into a binary variable indicating none versus suspected or definite psychotic-like experiences that were not attributable to sleep or fever.

#### Covariates

Covariates for epigenetic association analyses included child sex, maternal age at delivery (in years), prenatal maternal smoking (binary categorization of ‘no smoking/quit in early pregnancy’ vs. ‘smoked throughout pregnancy’), gestational age at delivery (in weeks), child age at MRI assessment (in years), total brain volume, technical covariates (i.e. batch effect in Generation R, surrogate variables in ALSPAC), and estimated cell-type proportions using a cord-blood-specific reference-based method (Gervin et al., 2019) in both cohorts and the Houseman method with the Reinius reference set (Houseman et al., 2012; Reinius et al., 2012) at 10 years in Generation R.

### Statistical analysis

#### Epigenome-wide association study

Linear regression was applied to investigate the prospective association between neonatal DNAm and LVV in childhood. In our EWAS analyses, the DNAm β value at each CpG site was specified as the predictor and total LVV as the outcome of interest, adjusted for all relevant covariates as described above. Probes were annotated using the *meffil* R package (Min et al., 2018), based on genome build hg19. *p*-values were adjusted for multiple testing using false discovery rate (FDR) adjustment (Benjamini & Hochberg, 1995) and only sites with FDR-corrected *q* < .05 were considered statistically significant.

**Figure 1:**
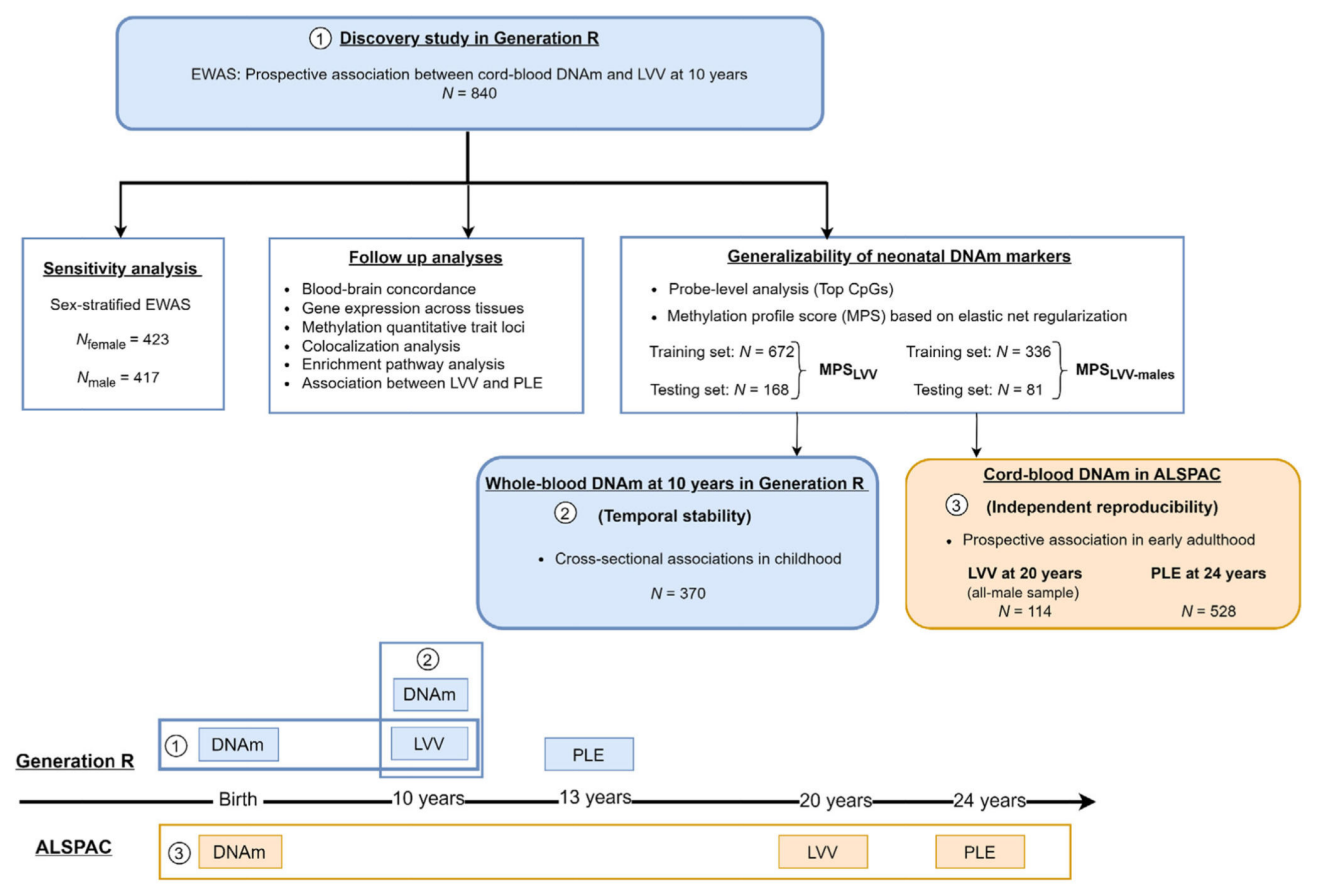
Summary flow chart of the analytical approach

Due to the known sex differences in LVV (Trimarchi et al., 2013) and the availability of MRI data only in males within ALSPAC, we performed a sex-stratified EWAS as a sensitivity analysis to provide a more direct comparison with the replication sample. A flow chart overview of the analytical approach in this study is presented in Figure 1.

#### Follow-up analyses

Follow-up analyses were performed to further characterize LVV-associated CpGs (FDR *q* < .05) as follows: (a) We used two independent online tools to test whether top CpG sites show concordance between peripheral blood and brain, based on live brain tissue from 12 epilepsy patients (IMAGE–CpG; https://han-lab.org/methylation/default/imageCpG) and postmortem brain tissue from 71 to 75 adult participants (https://epigenetics.essex.ac.uk/bloodbrain/); (b) Gene expression profiles were probed across 54 human tissues and in the developing brain over lifespan, using GTEx and BrainSpan data from the FUMA portal (https://fuma.ctglab.nl/); (c) Look-up of methylation quantitative trait loci (mQTL) was performed using the largest mQTL database to date to explore potential genetic influences on DNAm levels (https://mqtldb.godmc.org.uk/).

Given that DNAm of CpGs below the epigenome-wide significance threshold may also account for phenotype variation and improve prediction accuracy, LVV-associated CpGs with a suggestive *p* < 1 × 10^−4^ were taken forward to the following analyses. First, we assessed whether LVV-associated CpG sites are enriched for genetic liability to schizophrenia by performing colocalization analysis, a procedure that estimates the probability of a single shared variant. To this end, we first queried GoDMC (Min et al., 2021) to identify independent cis-mQTLs for the LVV-associated CpGs (*p* < 1 × 10^−8^, within 1 Mb from the CpG). When such mQTLs were identified, we extracted all available SNPs associations within a 1 Mb radius from these index mQTLs from GoDMC, and intersected with SNPs from a GWAS of schizophrenia (Lam et al., 2019). We excluded CpG sites with less than 10 mQTLs. On the resulting data, a Bayesian colocalization analysis was performed using the *coloc*.*abf* function with default skeptical priors from the *coloc* R package (Giambartolomei et al., 2018; Wallace, 2020). A posterior probability of a single shared variant (PP_H4_) ≥.8 was considered sufficient evidence for colocalization (see Appendix S1 for further details). Second, pathway enrichment analysis was performed based on Gene Ontology (GO) and the Kyoto Encyclopedia of Genes and Genomes (KEGG) using the *gometh* function of the *missMethyl* R package (Phipson, Maksimovic, & Oshlack, 2016). Independent pathways with FDR *q* < .05 were considered significant.

Last, to verify the relevance of childhood LVV to psychotic outcomes in the pediatric population, we tested whether LVV at age of 10 years was prospectively associated with hallucinations (categorical outcome using ordinal logistic regression analysis) and delusions (dimensional outcome using multivariable linear regression analyses) later in adolescence. Analyses were adjusted for sex, age of psychotic-like experiences assessment, ethnicity, and total brain volume.

#### Generalizability of neonatal DNAm markers associated with LVV. Temporal stability from birth to childhood in Generation R

Using the repeated measures of epigenetic data in Generation R, we examined whether the DNAm sites identified at birth remained associated with childhood LVV when measured again using peripheral blood at age 10 years. To this end, we tested whether DNAm levels for top CpG sites (FDR *q* < .05) correlated between time points (birth and age 10), and whether they were cross-sectionally associated with LVV (i.e. both DNAm and LVV assessed concurrently at age of 10 years) based on linear regression analyses.

In addition to testing single top CpGs, we also applied a MPS approach to examine associations using a broader epigenetic profile of LVV. Specifically, we first preselected LVV-associated CpGs at *p* < 1 × 10^−4^ from the cord blood EWAS. We then split the cord blood DNAm sample into training (80%, *N* = 672) and testing (20%, *N* = 168) datasets using the *createDataPartition* function in the *caret* R package (Kuhn, 2008), accounting for even distribution of LVV in the training and testing datasets. The preselected CpGs were used to establish the predictive model and feature selection in the training dataset using elastic net regularization (ENR), a machine-learning approach. The advantage of ENR is selecting informative features without compromising prediction accuracy (Zou & Hastie, 2005). Optimal combinations of the mixing (alpha) and shrinkage (lambda) parameters were determined via 10-fold cross-validations implemented in the *cva*.*glmnet* function of *glmnetUtils* package (Friedman, Hastie, & Tibshirani, 2010).

CpGs with non-zero coefficients from the elastic net model with the optimal alpha and lambda values were extracted and used as external weights to construct MPS in the testing set, which enabled us to evaluate direct replication and prediction performance. Specifically, an ENR-weighted DNAm sum score for LVV (i.e. MPS_LVV_) was calculated by multiplying the methylation value at a given CpG by the ENR estimated weight, and then summing these values: MPS_LVV_ = β1*CpG1 + β2*CpG2 ... + βi*CpGi. To assess the incremental utility of the MPS_LVV_ over and above covariates, we estimated the incremental *R*^2^ by comparing the predictive performance of the full model including the MPS_LVV_ to that of the covariate-only model.

Next, the ENR estimated weights for selected CpGs were used to construct MPS_LVV_childhood_ using whole blood DNAm at 10 years in Generation R. We then examined the cross-sectional association between MPS_LVV_childhood_ and LVV in childhood.

#### Generalizability of cord blood epigenetic associations with adult LVV in ALSPAC

Finally, we explored the generalizability of our findings in an independent all-male sample (*N* = 114) with complete data on cord blood DNAm at birth and MRI at age of 18–21 from ALSPAC. Again, we examined both the individual top CpGs as well as the MPS (constructed using cord blood DNAm in ALSPAC). In addition to the MPS_LVV_ derived from the discovery of birth EWAS in the overall sample (*N*_Generation R_ = 840), the same ENR approach was repeated to construct a MPS_LVV-males_ in ALSPAC, based on the all-male EWAS (*N*_Generation R-males_ = 417) to maximize comparability with the characteristics of the replication sample. We estimated how much variance (incremental *R*^2^) in LVV and psychotic-like experiences was explained by the ENR-based MPS_LVV-males_ and MPS_LVV_, respectively. Similar covariates (e.g. different ages of outcome assessments and surrogate variables in ALSPAC) were included in regression models as in Generation R.

In addition, we performed a logistic regression model to investigate whether LVV at age of 20 is associated with psychotic-like experiences at age of 24 in ALSPAC, after adjusting for total brain volume and age of outcome assessment.

## Results

Sample characteristics are described in Table 1. We compared children included in our selected EWAS sample (*N* = 840) with children who had complete data on LVV and covariates but not on neonatal DNAm (*N* = 1,928). This showed that children in the analytical sample on average were born later, had mothers who were older, and had a larger total brain volume. No differences were found for child sex, maternal education, smoking during pregnancy, and LVV.

### Epigenome-wide association study

**Figure 2:**
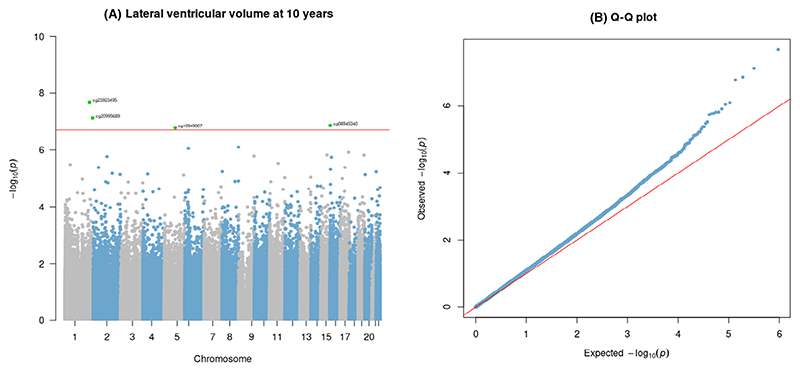
Manhattan (A) and quantile-quantile (B) plots showing genome-wide associations between DNAm at birth and LVV at age of 10 years in Generation R (*N* = **840**), λ = 1.16

At birth, four CpGs prospectively associated with childhood LVV after FDR correction (*q* < .05; Table 2 and Figure 2A), including (a) cg23923495 located in the gene body of *KCTD3* (potassium channel tetramerization domain containing 3); (b) cg20995689, located in the gene body of *SDCCAG8* (SHH signaling and ciliogenesis regulator); (c) cg08945340, located at an intergenic site that is not annotated to any genes; and (d) cg10949007, annotated to a promoter region of *GLRX* gene (glutaredoxin). Suggestive associations (*p* < 1 × 10^−5^) were observed at 26 additional CpG sites (Table 2). There was no evidence of genomic inflation in the EWAS (λ = 1.16, see the quantile–quantile plot Figure 2B).

One of these four probes, cg10949007, also remained significant after FDR correction in the all-male EWAS (*N* = 417). Two additional CpGs, cg22874802 and cg23737062 mapped to the *EPHA2* and *FBXL22* genes were identified after FDR correction in the male sample. No CpGs reached genome-wide significance in females, despite using a comparable sample size (*N* = 423). Detailed results of the sex-stratified EWASs are shown in Table S1 and Figure S2.

### Follow-up analyses

#### Blood–brain concordance

IMAGE–CpG using live brain tissue showed that DNAm in blood at cg08945340 was highly and positively correlated with DNAm in brain tissue (*r* = .62, Table S2), whereas the other two sites showed negative correlations (cg10949007, *r* = −.30; cg20995689, *r* = −.40), suggesting moderate inverse methylation patterns between blood and brain. A weaker correlation pattern for all four CpGs was observed when using the Blood Brain DNA Methylation Comparison Tool (*r*_*s*_ = −.16–.23; Table S2), with cg23923495 showing the highest correlation between blood and prefrontal cortex (*r* = .23).

#### Gene expression analysis across tissues and across brain developmental stages

Next, we assessed gene expression levels across 54 tissues including blood and several brain regions from public GTEx data (Figure S3a). Generally, *KCTD3, SDCCAG8*, and *GLRX* were expressed across multiple tissues, including all the brain regions. In particular, both *KCTD3* and *GLRX* were highly expressed in the cerebellum of the brain, while *SDCCAG8* showed similar levels of expression in all the brain regions. Data from BrainSpan further indicated that *KCTD3* and *SDCCAG8* were moderately to highly expressed during the prenatal period, decreasing in expression after birth. In contrast, *GLRX* showed higher expression in adulthood compared with other developmental stages (Figure S3b).

#### Methylation quantitative trait loci

Of the four significant CpGs identified at birth, cg10949007 in *GLRX* and cg08945340 were associated with 78 known mQTLs (*p* < 1 × 10^−8^, Table S3). All the identified associations were in *cis*.

#### Colocalization analysis

Using a total of 140 LVV-associated CpGs at *p* < 1 × 10^−4^, we tested for the presence of shared genetic variants with schizophrenia GWAS loci. We identified 98 independent cis-mQTLs at *p* < 1 × 10^−8^ from GoDMC. After intersecting with schizophrenia genetic variants within a 1 Mb radius, 57 CpG sites remained with a sufficient number of SNPs (≥10) for colocalization analysis (Table S4). One CpG site (cg08170519) annotated to *IGSF9B* showed strong evidence of colocalization (PP_H4_ = .98), suggesting a single shared variant affecting the two traits (i.e. LVV-associated DNAm and schizophrenia).

#### Pathway enrichment analysis

After removing all probes containing an SNP and cross-reactive probes, a total of 114 CpGs at *p* < 1 × 10^−4^ were annotated to 86 genes. GO and KEGG analyses yielded no significantly enriched common biological processes, cellular components, or molecular functions after FDR correction (*q* < .05). The top GO terms and KEGG pathways are included in Table S5a–b.

#### Associations between LVV and psychotic-like experiences

In Generation R, LVV at age of 10 years was not prospectively associated with self-reported hallucinations (*OR* = .98, 95% *CI* = [0.85; −1.13], *p* = .82), but a larger LVV was related to more delusions (*B* = .05, *SE* = .02; *p* = .047) at age of 14 years.

### Generalizability of neonatal DNAm markers associated with LVV

We performed ENR to select informative CpG features for constructing the MPS of LVV. First, we preselected the 140 CpGs significant at *p* < 1 × 10^−4^ from the discovery EWAS based on the overall sample at birth. Cross-validation identified a panel of 125 out of 140 preselected CpGs with an optimal combination of alpha (0.027) and lambda (0.170) in the training set. A MPS_*LVV*_ based on the ENR estimated weights was strongly predictive of LVV in the testing set (incremental *R*^2^ = 0.22). Detailed results of all MPS analyses are shown in Table 3.

To construct the male-specific MPS_LVV_males_, a total of 136 CpGs were preselected at *p* < 1 × 10^−4^ from the all-male EWAS performed at birth. An optimal combination of alpha (0) and lambda (.276) included all 136 preselected CpGs in the training set (*N* = 336). The MPS_LVV_males_ showed an excellent prediction of LVV in the testing set (incremental *R*^2^ = .33).

These ENR-estimated nonzero coefficients were then used as external weights to construct: (a) an MPS_LVV_childhood_ (using whole blood DNAm at 10 years) in Generation R to test cross-sectional associations with LVV, and (b) an MPS_LVV_ and an MPS_LVV_males_ (using cord blood DNAm at birth) in an independent sample of older males in ALSPAC, to test prospective associations with LVV in young adulthood.

#### Temporal stability from birth to childhood in generation R

The correlations between DNAm levels at the four top sites across time points (i.e. at birth vs. childhood) were low, ranging from −0.04 to 0.18 (Table S6a), which suggests high variability over time. No nominally significant cross-sectional associations at 10 years were observed for these individual CpG sites (Table S6b). In contrast to the single probe analysis, the MPS_LVV_childhood_ was significantly associated with LVV (β = .19, SE = .06, *p* = .001), explained 2.6% of the variance of LVV in the childhood DNAm sample.

#### Generalizability of cord blood epigenetic associations with adult LVV in ALSPAC

As a final step, we tested the generalizability of our findings in ALSPAC using LVV measured at age of 20 years. None of the four top CpG sites at birth reached statistical significance in the probe-level analysis, with three sites showing a consistent direction of associations (Table S7a,b). However, the male-specific MPS_LVV_males_ significantly associated with LVV in this all-male adult sample, explaining 3.9% of the variance in LVV on top of all covariates (β = .34, SE = .15, *p* = .02). The model with MPS_LVV_ (based on a discovery EWAS using both males and females) explained 3% of the variance in this phenotype (β = −.30, SE = .14, *p* = .04) but in a negative direction.

No significant association between the MPS_LVV_ and psychotic-like experiences was observed in ALSPAC (β = −13.17, SE = 7.63, *p* = .08). We also did not find a prospective association between LVV and psychotic-like experiences in the all-male sample (β = .26, SE = .27, *p* = .33).

## Discussion

To our knowledge, this is the first study to investigate prospective associations between epigenome-wide DNAm at birth and LVV in childhood, a key brain feature implicated in risk for schizophrenia. We identified four CpGs at birth that were prospectively associated with variation in LVV at age of 10 years, based on data from over 800 participants. These sites map to genes that have been implicated in brain development and psychiatric disorders including schizophrenia, pointing to interesting candidates for future investigation. None of these four CpGs showed temporally stable associations in childhood (using repeatedly assessed DNAm in Generation R) or were found to prospectively associate with LVV in an independent sample of young male adults (ALSPAC), likely due to the small effect sizes of these single CpGs in combination with the use of smaller sample sizes for these analyses. Notably, however, the use of a broader MPS of LVV including a much larger selection of CpGs led to improved predictive power, showing significant cross-sectional associations with LVV in childhood in Generation R (i.e. temporal stability) and significant prospective associations with LVV in young adulthood in ALSPAC (reproducibility). We also found evidence for the colocalization of genetic variants associated with LVV-related CpGs and schizophrenia. In the future, the use of larger multi-cohort analyses and replication efforts (Rijlaarsdam et al., 2016) with neuroimaging data collected at more comparable time points will be important in order to further elucidate the role of these DNAm patterns in LVV development and related risk for schizophrenia, and what factors explain the identified DNAm profile of LVV at birth.

### DNA methylation patterns at birth prospectively associate with LVV in childhood

Our EWAS at birth identified four CpGs after genome-wide correction, three of which were annotated to genes. The top site (cg23923495) is located in the gene body of *KCTD3*, a gene encoding the KCTD (potassium channel tetramerization domain) family protein that is a major regulator of cAMP signaling, which is essential for neuronal function (Muntean et al., 2022). *KCTD3* is widely expressed in the brain and has been involved in several neurocognitive and neurodevelopmental disorders, including cerebellar hypoplasia and autism (Marshall et al., 2008; Teng et al., 2019).

*SDCCAG8* (cg20995689), a centrosome-associated protein-encoding gene, is involved in neuronal migration in the developing cortex (Insolera, Shao, Airik, Hildebrandt, & Shi, 2014) and normal cilia formation and function (Flynn, Whitton, Donohoe, Morrison, & Morris, 2019). Studies in animals have shown that *SDCCAG8* expression associates with enlarged lateral ventricle in mice (Insolera et al., 2014). *SDCCAG8* is highly expressed in the human fetal brain during development (Figure S3b), and alterations in *SDCCAG8* expression have been suggested as a candidate pathway underlying risk for schizophrenia and related cognitive deficits (Flynn et al., 2019). Furthermore, existing GWASs have linked genetic variation in *SDCCAG8* with several brain-related phenotypes at genome-wide significant levels, including schizophrenia (Lam et al., 2019), educational attainment (Okbay et al., 2022), cognitive performance (Lam et al., 2017), and risk-taking behavior (Karlsson Linnér et al., 2019). A recent EWAS has also linked DNAm of *SDCCAG8* with prenatal maternal stressful life events associated with risk of schizophrenia (Kotsakis Ruehlmann et al., 2023).

*GLRX* (cg10949007) is a gene encoding, a member of the glutaredoxin family. This protein plays a protective role in nerve cell function in the presence of oxidative stress, by catalyzing the reversible reduction of glutathione (GSH), a major antioxidant. Previous studies found that lower GSH levels in the blood predict cognitive deficits and brain volume loss in children and adolescents with first-episode psychosis at 2 years of follow-up (Fraguas et al., 2012; Martínez-Cengotitabengoa et al., 2014). *GLRX* has been associated with autism, cognitive aging in healthy people and amyloid-β neuropathology in Alzheimer’s disease (Bowers et al., 2011; Harris et al., 2007; Wang et al., 2020). Altogether, these results suggest that cord blood DNAm sites, which were prospectively associated with childhood LVV, are annotated to genes involved in brain function, neural development, and psychiatric risk, including schizophrenia.

Colocalization analyses were performed to test whether CpGs in cord blood associated with LVV potentially share genetic variants with schizophrenia. One CpG site showed significant colocalization: cg08170519 is located in *IGSF9B*, a gene involved in GABA neurotransmission/inhibitory synapse development and Vitamin D receptor pathway processes, which have been repeatedly implicated in schizophrenia (Cui, McGrath, Burne, & Eyles, 2021). Our findings on colocalization and mQTLs show that genetic factors might implicate in the identified DNAm patterns. In future, the integration of genetic influences with prenatal environmental factors may provide a deeper insight linking early life epigenetics, LVV, and genetic liability to schizophrenia.

### Cord blood epigenetic profile of LVV persists into childhood

Interestingly, none of the top hits identified at birth (i.e. prospective analysis) continued to associate with LVV when DNAm was measured concurrently to MRI scans at 10 years (i.e. cross-sectional analysis). However, a broader MPS_LVV_childhood_ containing 125 CpG sites selected based on ENR was found to cross-sectionally associate with LVV in childhood (2.6% of variance explained on top of covariates), supporting a degree of temporal stability in DNAm patterns associated with LVV.

Previous longitudinal epigenetic studies have found that DNAm at birth is more strongly predictive of certain neurodevelopmental outcomes, such as ADHD (Neumann et al., 2020), compared with DNAm patterns examined later in childhood. Our findings demonstrated that MPS capturing broader DNAm patterns might be more stable and reproducible across different time points than the analysis of single CpG sites.

### A MPS at birth prospectively associates with LVV in young adulthood in an independent cohort

We further showed that a male-specific MPS_LVV_males_ derived from a smaller but more comparable discovery EWAS (i.e. including only the 417 males in Generation R, not the females) prospectively associated with LVV measured later in life (at age of 20 years instead of age 10 years in the discovery sample), within in an independent sample of young adults from ALSPAC, explaining 3.9% of variance in this phenotype over and above covariates. Interestingly, we found that our prediction performed better when using this male-specific MPS_LVV–males_, compared to an MPS_LVV_ using results from a larger discovery EWAS (i.e. from the full sample of 840 individuals comprising both males and females), pointing to the importance of maximizing comparability between the characteristics of the discovery EWAS and the replication sample. Of note, we were not able to replicate individual top CpGs in ALSPAC, likely due to both the small effect sizes observed and the small sample size of the replication sample (with corresponding larger standard errors). Overall, these findings further support the use of MPSs for capturing a broader epigenetic profile associated with LVV with improved predictive power and reproducibility.

Of note, while we found that LVV in childhood prospectively associated with self-reported delusions later in adolescence in Generation R, this prospective association was not identified at an older age in ALSPAC, which could reflect the potential functional significance of LVV in early development or differences in measurement of psychotic-like experiences (self-report vs. semi-structure interview). It should be noted that ALSPAC is the only other cohort (except for Generation R) to our knowledge with data on DNAm at birth and MRI collected in late childhood or adolescence. This prospective cohort allowed us to link cord blood-specific DNAm markers with LVV and related psychotic outcomes in early adulthood, another critical period for brain development and the onset of schizophrenia. Given that both DNAm and brain features vary substantially over time, it is crucial to evaluate the generalizability of epigenetic markers of LVV in a developmental context. More research using repeated measures of both DNAm and neuroimaging is needed to better understand their dynamic associations across development, as currently such datasets are extremely scarce.

### Strengths and limitations

The Generation R Study is a large birth cohort with prospectively collected DNAm and neuroimaging data on a population level, which enabled us to conduct prospective associations linking early epigenetic variation to later LVV. Other strengths of this study include the use of an epigenome-wide approach with a brain structure highly relevant for schizophrenia (i.e. LVV as an intermediate phenotype), the use of an independent prospective birth cohort to test the generalizability of our findings, and the use of MPSs based on a machine learning approach (i.e. ENR) to capture a broader epigenetic profile of LVV and help improve predictive power. In addition, the availability of DNAm at multiple time points enabled us to explore developmental aspects of the association between DNAm and LVV, although we cannot separate true temporal signals from other potential contributing factors such as cell-type composition differences in blood at these different time points.

Our findings must also be interpreted in the context of several limitations. First, the sample sizes and measurement of psychotic-like experience varied between the two cohorts examined, which limited our ability to test the generalizability of findings. Second, the psychotic-like experiences were self-reported by adolescents in Generation R, which may be subject to reporting bias because of a misinter-pretation of the questions. Nevertheless, there is evidence from longitudinal population studies that self-report assessments of psychotic-like experiences during early adolescence are predictive for both psychotic-like experiences and clinical diagnosis later in adulthood (Healy et al., 2019; Isaksson, Angenfelt, Frick, Olofsdotter, & Vadlin, 2022). Third, the 450 k array only covers 1.7% of the total number of CpGs in the human genome, compared with the EPIC array targeting approximately 850,000 sites (3% of total CpGs). Furthermore, selected CpG sites are substantially biased toward promoter and gene body regions, which limits the capacity to detect potential associations between DNAm with LVV at other unmeasured loci. Fourth, the study focused exclusively on DNAm. Other epigenetic processes (e.g. histone modifications or circulating microRNAs) are likely to also be important for brain phenotypes. Last, despite the fact that we identified prospective associations between DNAm and LVV, it is not possible to establish causality.

## Conclusion

We conducted the first EWAS of DNAm at birth with LVV in childhood – a brain feature robustly linked to schizophrenia risk by previous research and found to prospectively associate with adolescent self-reported delusions in our population-based cohort. We identified several genome-wide significant CpG sites that map to genes implicated in brain development and the brain-related traits, including schizophrenia. A MPS based on the EWAS results at birth was found to cross-sectionally associate with LVV when measured in childhood (showing evidence of temporal stability), and to prospectively associate with LVV in young adulthood within an independent cohort of older males (showing evidence of reproducibility). Prediction in this independent sample was stronger and more robust when the MPS was based on male-specific EWAS results compared with the sex-combined EWAS results, highlighting the importance of maximizing comparability between the characteristics of the discovery and replication samples. Future studies with larger samples, repeated measures of DNAm, and MRI, more comparable measurement time-points between studies and better epigenomic coverage are needed to confirm our findings, and integrated genetic and prenatal environmental factors to elucidate which factors explain variability in LVV-associated epigenetic patterns at birth and downstream risk for schizophrenia.

## Supplementary Material

Appendix

Supplementary material

## Figures and Tables

**Table 1:** Study population characteristics

		ALSPAC	
Child characteristics	Generation R (*N* = 840)	MRI subsample (*N* = 114)	PLE subsample (*N* = 528)
Sex, *N* (%), female	423 (50.4)	0 (0.0)	312 (59.1)
Gestational age, mean (*SD*), weeks	40.2 (1.5)	39.5 (1.6)	39.6 (1.5)
Maternal age at delivery, mean (*SD*), years	32.0 (4)	29.8 (4.4)	29.9 (4.3)
Paternal age at intake, mean (*SD*), years	34.3 (4.9)	NA^a^	NA^a^
Sustained smoking during pregnancy, *N* (%), yes	88 (10.5)	11 (10.1)	48 (9.7)
Education, *N* (%), higher education	574 (67.8)	30 (26.3)	130 (24.6)
Age at MRI assessment, mean (*SD*), years	10.1 (0.6)	19.5 (0.8)	20.1 (1.3)
Total volume of lateral ventricles, mean (*SD*), cm^3^	11,015.7 (6,140.4)	13,889.0 (7681.9)	12,430.2 (6,504.5)
Total brain volume, mean (*SD*), cm^3^	1,235,070 (106,535.3)	1,779,899 (129,775.9)	1,748,234 (156,801.0)
Age at PLE assessment, mean (*SD*), years	13.55 (0.37)	24.4 (0.8)	24.4 (0.7)
Psychotic-like experiences, yes, *N* (%)		8 (10.1)	44 (8.3)
Delusions, mean (*SD*)^b^	0.9 (1.35)		
Hallucinations, *N* (%)^c^			
None	2062 (87)		
Moderate	258 (11)		
Severe	40 (2)		

a The mean age of fathers at intake (30.38 years) was only available in the overall sample in ALSPAC.

b *N*_delusions_ = 1899.

c *N*_hallucinations_ = 2,360 in Generation R.

**Table 2:** CpG sites in cord blood suggestively associated (*p* < 1 × 10^−5^) with LVV at age of 10 years in Generation R (*N* = 840)

CpG site	Chromosome	Position	Nearest gene	Beta	SE	*p*-value	FDR
cg23923495	1	215741843	*KCTD3*	–.259	.046	2.08E–08	.010
cg20995689	1	243647204	*SDCCAG8*	–.252	.046	7.51E–08	.018
cg08945340	16	1077028	NA	–.218	.041	1.37E–07	.020
cg10949007	5	95159614	*GLRX*	.205	.039	1.68E–07	.020
cg21511395	8	144942223	*EPPK1*	–.244	.049	8.00E–07	.070
cg18950108	6	30920171	*DPCR1*	–.223	.045	8.87E–07	.070
cg11025757	17	73738713	*ITGB4*	–.210	.043	1.22E–06	.072
cg08033551	15	74659966	*CYP11A1*	–.202	.042	1.52E–06	.072
cg04865692	19	50831762	*KCNC3*	–.191	.040	1.53E–06	.072
cg13860266	9	136787087	*VAV2*	–.200	.041	1.66E–06	.072
cg19102412	2	121224327	*LOC84931*	–.278	.058	1.73E–06	.072
cg02535219	16	11876908	*ZC3H7A*	–.194	.040	1.82E–06	.072
cg15526825	11	75237636	*GDPD5*	.160	.034	2.98E–06	.108
cg16710124	1	44716058	*ERI3*	–.254	.054	3.30E–06	.112
cg12812662	17	41857603	*DUSP3;C17orf105*	–.264	.057	4.16E–06	.124
cg12485219	2	46588351	*EPAS1*	–.248	.054	4.17E–06	.124
cg06723414	16	3763022	*TRAP1*	–.232	.050	4.85E–06	.135
cg02083412	8	1649758	*DLGAP2*	–.230	.050	5.84E–06	.148
cg18262937	22	17002291	NA	–.168	.037	5.92E–06	.148
cg11369761	2	168151051	NA	–.166	.037	6.70E–06	.153
cg01166925	4	41880280	NA	–.166	.037	7.09E–06	.153
cg00573606	2	238322680	*COL6A3*	–.212	.047	7.34E–06	.153
cg08284598	20	3218145	*SLC4A11*	–.231	.051	7.42E–06	.153
cg21503476	5	169531595	*FOXI1*	–.235	.052	7.73E–06	.153
cg12314335	13	113699016	*MCF2L*	–.192	.043	9.17E–06	.172
cg08170519	11	133804959	*IGSF9B*	–.174	.039	9.45E–06	.172

NA, not available. The full model is adjusted for batch effects, estimated cell-type proportions, child sex, gestational age, maternal age in take/delivery, maternal smoking during pregnancy, child age at MRI assessment and total brain volume.

**Table 3:** Generalizability with methylation profile scores for LVV

ENR-based MPS			*N* of CpGs	β	SE	*p*-value	*R*^2^_basic model	*R*^2^_MPS model	*R*^2^ change
Generation R									
Cord-blood DNAm testing sample (*N* = 168)									
MPS_LVV_		125		.420	.064	1.37E–09	.115	.333	.218
Cord-blood DNAm all-male testing sample (*N *= 81)									
MPS_LVV_males_		136		.581	.105	1.70E–06	.180	.511	.330
Childhood whole blood DNAm sample (*N *= 370)									
MPS_LVV_childhood_	125			.188	.058	.001	.066	.092	.026
ASLPAC (Cord-blood DNAm)									
LVV all-male sample (*N* = 114)									
MPS_LVV_	125			–.296	.143	.040	–.010	.020	.030
MPS_LVV___males_	136			.338	.147	.024	–.010	.028	.039
PLE sample (*N* = 528)									
MPS_LVV_	125			–13.172	7.629	.084	.527	.697	.170
MPS_LVV___males_	136			–.888	1.110	.423	.527	.536	.010

Regression results of ENR-based MPS on LVV at 10 years in Generation R, LVV at 20 years and PLE at 24 years in ALSPAC. Regression models are corrected for relevant covariates as in EWAS analysis. ENR-based MPS: using elastic net regularization, the MPS_LVV_ was constructed based on ENR estimated weights using preselected CpGs from EWAS in the overall sample, MPS_LVV_males_ was constructed based on those from the all-males EWAS.

PLE, psychotic-like experiences. *R*^2^: Adjusted *R*-squared. *R*^2^ change was calculated by building a basic model with covariates only (without MPS) and then a MPS model that included MPS as a predictor. The *R*^2^ of the basic model was subtracted from those of the MPS model.

## Data Availability

The summary statistics of the EWAS in the overall sample and the sex-stratified EWAS, the ENR-based weights of the selected CpGs are available under license CC BY 4.0, with the identifier: https://doi.org/10.6084/m9.figshare.22434637.v1.

## Key points

- We employed an epigenome-wide, longitudinal design in a large population-based birth cohort to study epigenetic correlates of lateral ventricular volume (LVV), a known correlate of schizophrenia risk.
- Several genome-wide significant CpG sites at birth were prospectively associated with LVV at age of 10, which in turn related to self-reported delusions at age of 14. Identified loci map to genes previously implicated in brain development and psychiatric disorders, including schizophrenia.
- A methylation profile score (MPS) based on the epigenome-wide association study (EWAS) results was found to associate with LVV cross-sectionally when measured from blood at age of 10, supporting a degree of temporal stability in DNAm–LVV associations.
- A male-specific MPS at birth also predicted LVV in early adulthood using an independent, older cohort of males. These associations were more robust and stronger than using a sex-combined MPS, which highlights the importance of maximizing comparability between the characteristics of the discovery EWAS and the replication sample.

## References

[R1] Aas M, Haukvik UK, Djurovic S, Bergmann Ø, Athanasiu L, Tesli MS, Melle I (2013). BDNF val66met modulates the association between childhood trauma, cognitive and brain abnormalities in psychoses. Progress in Neuro-Psychopharmacology and Biological Psychiatry.

[R2] Adriaanse M, van Domburgh L, Zwirs B, Doreleijers T, Veling W (2015). School-based screening for psychiatric disorders in Moroccan-Dutch youth. Child and Adolescent Psychiatry and Mental Health.

[R3] Benjamini Y, Hochberg Y (1995). Controlling the false discovery rate: A practical and powerful approach to multiple testing. Journal of the Royal Statistical Society: Series B (Methodological).

[R4] Birnbaum R, Weinberger DR (2017). Genetic insights into the neurodevelopmental origins of schizophrenia. Nature Reviews Neuroscience.

[R5] Bolhuis K, Koopman-Verhoeff ME, Blanken LME, Cibrev D, Jaddoe VWV, Verhulst FC, Tiemeier H (2018). Psychotic-like experiences in pre-adolescence: What precedes the antecedent symptoms of severe mental illness?. Acta Psychiatrica Scandinavica.

[R6] Bolhuis K, Tiemeier H, Jansen PR, Muetzel RL, Neumann A, Hillegers MHJ, Kushner SA (2019). Interaction of schizophrenia polygenic risk and cortisol level on pre-adolescent brain structure. Psychoneuroendocrinology.

[R7] Bowers K, Li Q, Bressler J, Avramopoulos D, News-chaffer C, Fallin MD (2011). Glutathione pathway gene variation and risk of autism spectrum disorders. Journal of Neurodevelopmental Disorders.

[R8] Boyd A, Golding J, Macleod J, Lawlor DA, Fraser A, Henderson J, Smith GD (2013). Cohort profile: The ’Children of the 90s’-the index offspring of the Avon longitudinal study of parents and children. International Journal of Epidemiology.

[R9] Cannon TD, Mednick SA, Parnas J, Schulsinger F, Praestholm J, Vestergaard A (1993). Developmental brain abnormalities in the offspring of schizophrenic mothers. 1. Contributions of genetic and perinatal factors. Archives of General Psychiatry.

[R10] Cecil CAM, Neumann A, Walton E (2023). Epigenetics applied to child and adolescent mental health: Progress, challenges and opportunities. JCPP Advances.

[R11] Chen J, Zang Z, Braun U, Schwarz K, Harneit A, Kremer T, Schwarz E (2020). Association of a reproducible epigenetic risk profile for schizophrenia with brain methylation and function. JAMA Psychiatry.

[R12] Cui X, McGrath JJ, Burne THJ, Eyles DW (2021). Vitamin D and schizophrenia: 20 years on. Molecular Psychiatry.

[R13] Dall’Aglio L, Muka T, Cecil CAM, Bramer WM, Verbiest MMPJ, Nano J, Tiemeier H (2018). The role of epigenetic modifications in neurodevelopmental disorders: A systematic review. Neuroscience & Biobehavioral Reviews.

[R14] Flynn M, Whitton L, Donohoe G, Morrison CG, Morris DW (2019). Altered gene regulation as a candidate mechanism by which ciliopathy gene SDCCAG8 contributes to schizophrenia and cognitive function. Human Molecular Genetics.

[R15] Fraguas D, Gonzalez-Pinto A, Micó JA, Reig S, Parellada M, Martínez-Cengotitabengoa M, Arango C (2012). Decreased glutathione levels predict loss of brain volume in children and adolescents with first-episode psychosis in a two-year longitudinal study. Schizophrenia Research.

[R16] Fraser A, Macdonald-Wallis C, Tilling K, Boyd A, Golding J, Davey Smith G, Lawlor DA (2013). Cohort profile: The Avon longitudinal study of parents and children: ALSPAC mothers cohort. International Journal of Epidemiology.

[R17] Friedman JH, Hastie T, Tibshirani R (2010). Regularization paths for generalized linear models via coordinate descent. Journal of Statistical Software.

[R18] Gervin K, Salas LA, Bakulski KM, van Zelm MC, Koestler DC, Wiencke JK, Jones MJ (2019). Systematic evaluation and validation of reference and library selection methods for deconvolution of cord blood DNA methylation data. Clinical Epigenetics.

[R19] Giambartolomei C, Zhenli Liu J, Zhang W, Hauberg M, Shi H, Boocock J, Roussos P (2018). A Bayesian framework for multiple trait colocalization from summary association statistics. Bioinformatics.

[R20] Harris SE, Fox H, Wright AF, Hayward C, Starr JM, Whalley LJ, Deary IJ (2007). A genetic association analysis of cognitive ability and cognitive ageing using 325 markers for 109 genes associated with oxidative stress or cognition. BMC Genetics.

[R21] Healy C, Brannigan R, Dooley N, Coughlan H, Clarke M, Kelleher I, Cannon M (2019). Childhood and adolescent psychotic experiences and risk of mental disorder: A systematic review and meta-analysis. Psychological Medicine.

[R22] Houseman EA, Accomando WP, Koestler DC, Christensen BC, Marsit CJ, Nelson HH, Kelsey KT (2012). DNA methylation arrays as surrogate measures of cell mixture distribution. BMC Bioinformatics.

[R23] Insolera R, Shao W, Airik R, Hildebrandt F, Shi S-H (2014). SDCCAG8 regulates pericentriolar material recruitment and neuronal migration in the developing cortex. Neuron.

[R24] Isaksson J, Angenfelt M, Frick MA, Olofsdotter S, Vadlin S (2022). Psychotic-like experiences from adolescence to adulthood: A longitudinal study. Schizophrenia Research.

[R25] Ivanova MY, Achenbach TM, Rescorla LA, Dumenci L, Almqvist F, Bilenberg N, Verhulst FC (2007). The generalizability of the youth self-report syndrome structure in 23 societies. Journal of Consulting and Clinical Psychology.

[R26] Kahn RS, Sommer IE, Murray RM, Meyer-Lindenberg A, Weinberger DR, Cannon TD, Insel TR (2015). Schizophrenia. Nature Reviews Disease Primers.

[R27] Karlsson Linnér R, Biroli P, Kong E, Meddens SFW, Wedow R, Fontana MA, Beauchamp JP (2019). Genome-wide association analyses of risk tolerance and risky behaviors in over 1 million individuals identify hundreds of loci and shared genetic influences. Nature Genetics.

[R28] Kaufman J, Birmaher B, Brent D, Rao U, Flynn C, Moreci P, Ryan N (1997). Schedule for affective disorders and schizophrenia for school-age children present and lifetime version (K-SADS-PL): Initial reliability and validity data. Journal of the American Academy of Child and Adolescent Psychiatry.

[R29] Kooijman MN, Kruithof CJ, van Duijn CM, Duijts L, Franco OH, Van IJzendoorn MH, Jaddoe VWV (2016). The generation R study: Design and cohort update 2017. European Journal of Epidemiology.

[R30] Kotsakis Ruehlmann A, Sammallahti S, Cortés Hidalgo AP, Bakulski KM, Binder EB, Campbell ML, Brunst KJ (2023). Epigenome-wide meta-analysis of prenatal maternal stressful life events and newborn DNA methylation. Molecular Psychiatry.

[R31] Kremen WS, Panizzon MS, Neale MC, Fennema-Notestine C, Prom-Wormley E, Eyler LT, Dale AM (2012). Heritability of brain ventricle volume: Converging evidence from inconsistent results. Neurobiology of Aging.

[R32] Kuhn M (2008). Building predictive models in R using the caret package. Journal of Statistical Software.

[R33] Kuo SS, Pogue-Geile MF (2019). Variation in fourteen brain structure volumes in schizophrenia: A comprehensive meta-analysis of 246 studies. Neuroscience & Biobehavioral Reviews.

[R34] Lam M, Chen C-Y, Li Z, Martin AR, Bryois J, Ma X, Huang H (2019). Comparative genetic architectures of schizophrenia in east Asian and European populations. Nature Genetics.

[R35] Lam M, Trampush JW, Yu J, Knowles E, Davies G, Liewald DC, Lencz T (2017). Large-scale cognitive GWAS meta-analysis reveals tissue-specific neural expression and potential nootropic drug targets. Cell Reports.

[R36] Lancaster K, Morris JP, Connelly JJ (2018). Neuroimaging epigenetics: Challenges and recommendations for best practices. Neuroscience.

[R37] Lehne B, Drong AW, Loh M, Zhang W, Scott WR, Tan S-T, Elliott P (2015). A coherent approach for analysis of the Illumina HumanMethylation450 BeadChip improves data quality and performance in epigenome-wide association studies. Genome Biology.

[R38] Marshall CR, Noor A, Vincent JB, Lionel AC, Feuk L, Skaug J, Scherer SW (2008). Structural variation of chromosomes in autism Spectrum disorder. The American Journal of Human Genetics.

[R39] Martínez-Cengotitabengoa M, Micó JA, Arango C, Castro-Fornieles J, Graell M, Payá B, González-Pinto A (2014). Basal low antioxidant capacity correlates with cognitive deficits in early onset psychosis. A 2-year follow-up study. Schizophrenia Research.

[R40] Min JL, Hemani G, Davey Smith G, Relton C, Suderman M (2018). Meffil: Efficient normalization and analysis of very large DNA methylation datasets. Bioinformatics.

[R41] Min JL, Hemani G, Hannon E, Dekkers KF, Castillo-Fernandez J, Luijk R, Suderman M (2021). Genomic and phenotypic insights from an atlas of genetic effects on DNA methylation. Nature Genetics.

[R42] Mulder RH, Neumann A, Cecil CAM, Walton E, Houtepen LC, Simpkin AJ, Suderman M (2021). Epigenome-wide change and variation in DNA methylation in childhood: Trajectories from birth to late adolescence. Human Molecular Genetics.

[R43] Mulder RH, Walton E, Neumann A, Houtepen LC, Felix JF, Bakermans-Kranenburg MJ, Cecil CAM (2020). Epigenomics of being bullied: Changes in DNA methylation following bullying exposure. Epigenetics.

[R44] Muntean BS, Marwari S, Li X, Sloan DC, Young BD, Wohlschlegel JA, Martemyanov KA (2022). Members of the KCTD family are major regulators of cAMP signaling. Proceedings of the National Academy of Sciences.

[R45] Neumann A, Walton E, Alemany S, Cecil C, González JR, Jima DD, Tiemeier H (2020). Association between DNA methylation and ADHD symptoms from birth to school age: A prospective meta-analysis. Translational Psychiatry.

[R46] Okbay A, Wu Y, Wang N, Jayashankar H, Bennett M, Nehzati SM, LifeLines Cohort S (2022). Polygenic prediction of educational attainment within and between families from genome-wide association analyses in 3 million individuals. Nature Genetics.

[R47] Olabi B, Ellison-Wright I, McIntosh AM, Wood SJ, Bullmore ET, Lawrie SM (2011). Are there progressive brain changes in schizophrenia? A meta-analysis of structural magnetic resonance imaging studies. Biological Psychiatry.

[R48] Orahilly R, Muller F (1990). Ventricular system and choroid plexuses of the human brain during the embryonic period proper. American Journal of Anatomy.

[R49] Phipson B, Maksimovic J, Oshlack A (2016). missMethyl: An R package for analyzing data from Illumina’s HumanMethylation450 platform. Bioinformatics.

[R50] Reinius LE, Acevedo N, Joerink M, Pershagen G, Dahlén S-E, Greco D, Kere J (2012). Differential DNA methylation in purified human blood cells: Implications for cell lineage and studies on disease susceptibility. PLoS One.

[R51] Richetto J, Meyer U (2021). Epigenetic modifications in schizophrenia and related disorders: Molecular scars of environmental exposures and source of phenotypic variability. Biological Psychiatry.

[R52] Rijlaarsdam J, Cecil CAM, Relton CL, Barker ED (2022). Epigenetic profiling of social communication trajectories and co-occurring mental health problems: A prospective, methylome-wide association study. Development and Psychopathology.

[R53] Rijlaarsdam J, Pappa I, Walton E, Bakermans-Kranenburg MJ, Mileva-Seitz VR, Rippe RC, Van IMH (2016). An epigenome-wide association meta-analysis of prenatal maternal stress in neonates: A model approach for replication. Epigenetics.

[R54] Sharp TH, McBride NS, Howell AE, Evans CJ, Jones DK, Perry G, Walton E (2020). Population neuroimaging: Generation of a comprehensive data resource within the ALSPAC pregnancy and birth cohort. Wellcome Open Research.

[R55] Smigielski L, Jagannath V, Rössler W, Walitza S, Grünblatt E (2020). Epigenetic mechanisms in schizophrenia and other psychotic disorders: A systematic review of empirical human findings. Molecular Psychiatry.

[R56] Teng X, Aouacheria A, Lionnard L, Metz KA, Soane L, Kamiya A, Hardwick JM (2019). KCTD: A new gene family involved in neurodevelopmental and neuropsychiatric disorders. CNS Neuroscience & Therapeutics.

[R57] Trimarchi F, Bramanti P, Marino S, Milardi D, Di Mauro D, Ielitro G, Cutroneo G (2013). MRI 3D lateral cerebral ventricles in living humans: Morphological and morphometrical age-, gender-related preliminary study. Anatomical Science International.

[R58] van Erp TGM, Hibar DP, Rasmussen JM, Glahn DC, Pearlson GD, Andreassen OA, Turner JA (2016). Subcortical brain volume abnormalities in 2028 individuals with schizophrenia and 2540 healthy controls via the ENIGMA consortium. Molecular Psychiatry.

[R59] Vojinovic D, Adams HH, Jian X, Yang Q, Smith AV, Bis JC, Fornage M (2018). Genome-wide association study of 23,500 individuals identifies 7 loci associated with brain ventricular volume. Nature Communications.

[R60] Wallace C (2020). Eliciting priors and relaxing the single causal variant assumption in colocalisation analyses. PLoS Genetics.

[R61] Walton E, Baltramonaityte V, Calhoun V, Heijmans BT, Thompson PM, Cecil CAM (2023). A systematic review of neuroimaging epigenetic research: Calling for an increased focus on development. Molecular Psychiatry.

[R62] Wang H, Yang J, Schneider JA, De Jager PL, Bennett DA, Zhang HY (2020). Genome-wide interaction analysis of pathological hallmarks in Alzheimer’s disease. Neurobiology of Aging.

[R63] Wheater ENW, Stoye DQ, Cox SR, Wardlaw JM, Drake AJ, Bastin ME, Boardman JP (2020). DNA methylation and brain structure and function across the life course: A systematic review. Neuroscience and Biobehavioral Reviews.

[R64] White T, Muetzel RL, El Marroun H, Blanken LME, Jansen P, Bolhuis K, Tiemeier H (2018). Paediatric population neuroimaging and the generation R study: The second wave. European Journal of Epidemiology.

[R65] Zammit S, Kounali D, Cannon M, David AS, Gunnell D, Heron J, Wolke D (2013). Psychotic experiences and psychotic disorders at age 18 in relation to psychotic experiences at age 12 in a longitudinal population-based cohort study. American Journal of Psychiatry.

[R66] Zou H, Hastie T (2005). Regularization and variable selection via the elastic net. Journal of the Royal Statistical Society: Series B (Statistical Methodology).

