# Supplementary material for: DNA methylation at birth and lateral ventricular volume in childhood: a neuroimaging epigenetics study
Source: J Child Psychol Psychiatry. Author manuscript; Available in PMC 2024 Apr 3. (PMC10953396; doi:10.1111/jcpp.13866)
Supplement: Appendix [file EMS194680-supplement-Appendix.docx]

**DNA Methylation at Birth and Lateral Ventricular Volume in Childhood:**

**A Neuroimaging Epigenetics Study**

**Supporting information**

**Appendix S1**

**Supplementary Methods**

# Generation R Study

***Study population***

The Generation R Study is a prospective population-based cohort (Kooijman et al., 2016). Pregnant women with an expected delivery date between April 2002 and January 2006 residing in the municipality of Rotterdam, the Netherlands, were invited to enroll in the study. In total, 9778 pregnant women had 9749 live-born children. The study has been approved by the Medical Ethical Committee of the Erasmus MC, University Medical Center Rotterdam (MEC 198.782/2001/31). Written informed consent was obtained for all participants.

***DNA methylation***

DNA was extracted from cord blood at birth and whole peripheral blood at 10 years using the salting-out method. 500 nanograms of DNA per sample underwent bisulfite conversion using the EZ-96 DNA Methylation kit (Shallow) (Zymo Research Corporation, Irvine, CA, USA). Samples were plated onto 96-well plates in no specific order. The bisulfite-converted DNA was then processed with the Illumina Infinium HumanMethylation450 (450k) BeadChip (Illumina Inc., San Diego, CA, USA). Quality control of analyzed samples was performed using standardized criteria. Quality control and normalization of the HumanMethylation450 BeadChip array data was performed according to the Control Probe Adjustment and reduction of global CORrelation (CPACOR) workflow using R (Lehne et al., 2015). Probes that had a detection p-value above background ≥ 1E-16 were set to missing per array. Next, the intensity values were quantile normalized for each of the six probe-type categories separately: type II red/green, type I methylated red/green, and type I unmethylated red/green. Beta values were calculated as the proportion of methylated intensity value to the sum of methylated and unmethylated intensities plus 100. Arrays with observed technical problems such as failed bisulfite conversion, hybridization or extension, as well as arrays with a sex mismatch were removed from subsequent analyses. Additionally, only arrays with a call rate > 95% per sample were processed further. Probes on the X and Y chromosomes were excluded from the analyses. The final datasets contained 457,774 probes in the newborn dataset and 458,563 probes in the 10-year-old dataset.

***Structural magnetic resonance images (MRI)***

Structural magnetic resonance images (MRI) were acquired at age 9‑11 years on a 3-Tesla MRI system (MR750w, General Electric, Milwaukee, WI, USA) using an 8-channel head coil. During each structural imaging session coronal T1 scans were collected. Imaging parameters were as follows: 3D fast spoiled gradient echo (FSPGR) with 168–182 oBetalique-axial AC-Precuneusslices, 1 mm isotropic resolution; flip angle = 20°; repetition time (TR) = 7.9 ms; echo time (TE) = 3.0 ms; inverse time (TI) = 450 ms; 1mm × 1mm x 1mm voxel size; slice thickness 1 mm; FOV (field of view) 256 × 192 mm matrix. T1- weighted scans took approximately 7.15 minutes each. Automatic volumetric segmentations of the structural T1-weighted images were processed using the FreeSurfer package, version 6.0 (Fischl et al., 2004). Detailed information on the imaging processing and quality assessment has been described previously (White et al., 2018).

***Psychotic-like experiences (PLEs)***

PLEs included hallucinatory and delusional experiences at age 14 years, respectively. The self-report hallucinations were assessed using two items from the Youth Self-Report (YSR): ‘I hear sounds or voices that are not there according to other people’ and ‘I see things that other people think are not there’ (Ivanova et al., 2007). Items were rated on a three-point scale: not at all (0), a bit (1), or clearly (2). Based on the sum scores, youth were classified into the following groups as used in previous studies: no symptoms (0 points), mild symptoms (score of 1 point on at least one of the items) and moderate-to-severe symptoms (score of 2 points on at least one of the items) (Bolhuis et al., 2018). Self-reported delusions were assessed using items derived from the Kiddie Schedule for Affective Disorders and Schizophrenia(K-SADS) (Adriaanse et al., 2015; Kaufman et al., 1997). The K-SADS is a validated diagnostic research tool for assessing psychiatric disorders in children and adolescents. Six items assess different types of delusions (e.g. ‘Have other people ever read your thoughts?’, ‘Have you ever believed that you were being sent special messages through television or radio?’, and ‘Have you ever believed that you are an important person or have special gifts other people do not have?’). Items were rated on a three-point scale: No (0), Yes, probably (1) and Yes, certainly (2), and summed into a total score of delusions as in previous studies (Adriaanse et al., 2015).

# Avon Longitudinal Study of Parents and Children (ALSPAC)

***Study population***

ALSPAC is a population-based prospective pregnancy cohort based in the United Kingdom. Pregnant women residing in the study area of former county Avon, United Kingdom, with an expected delivery date between 1^st^ April 1991 and 31^st^ December 1992 were invited to enroll in the ALSPAC study. The initial number of pregnancies enrolled is 14,541. Of these initial pregnancies, there was a total of 14,676 foetuses, resulting in 14,062 live births and 13,988 children who were alive at 1 year of age. For more information on the study design has been published previously (Boyd et al., 2013; Fraser et al., 2013). The ALSPAC study website contains details of all the data that are available through a fully searchable data dictionary and variable search tool (http://www.bristol.ac.uk/alspac/researchers/ourdata/). Study data were collected and managed using REDCap electronic data capture tools hosted at the University of Bristol.(Harris et al., 2009) REDCap (Research Electronic Data Capture) is a secure, web-based software platform designed to support data capture for research studies..Ethical approval for the study was obtained from the ALSPAC Ethics and Law Committee and the Local Research Ethics Committees. Consent for biological samples has been collected in accordance with the Human Tissue Act (2004). Informed consent for the use of data collected via questionnaires and clinics was obtained from participants following the recommendations of the ALSPAC Ethics and Law Committee at the time.

***DNA methylation***

DNA methylation was measured in cord blood from 1018 of these children as part of the Accessible Resource for Integrative Epigenomic Studies (ARIES) (Relton et al., 2015). Briefly, following DNA extraction samples were bisulphite converted using the Zymo EZ DNA Methylation™ kit (Zymo, Irvine, CA, USA). Following conversion, genome-wide methylation was measured using the Illumina Infinium HumanMethylation450 (HM450) BeadChip. Arrays were scanned using Illumina iScan (version 3.3.28). Quality control was performed using the meffil package in R version 3.4.3. Samples with mismatched genotypes, mismatched sex, incorrect relatedness, low concordance with samples collected at other time points, extreme dye bias and poor probe detection were removed. Full details of the pre-processing and normalisation has been described previously (Min et al., 2018).

***Structural magnetic resonance images (MRI)***

In ALSPAC, between the ages of 18 to 21 years, a subset of ALSPAC offspring were invited to participate in three different neuroimaging studies: the ALSPAC-Testosterone study, the ALSPAC-Psychotic Experiences (PE) study, and the ALSPAC- Schizophrenia Recall by Genotype (SCZ-RbG) study. In total, MRI data was acquired for 958 participants: 513 in the Testosterone study, 248 in the Psychotic Experiences study and 197 in the ALSPAC-Schizophrenia Recall by Genotype study^7^. The current study was based on the Testosterone study to maximize the sample size. High-resolution, T1-weighted structural MRI data were acquired using a coronal inversion recovery fast spoiled gradient recalled sequence with the following parameters: GE option BRAVO, TR = 8.77 ms, TE = 3.4 ms, TI = 600 ms, flip angle = 10°, matrix size = 220 × 220, field of view = 220 mm × 220 mm, slice thickness = 1 mm, number of slices = 230, ARC acceleration factor = 2. Processing steps and details have been described elsewhere (Sharp et al., 2020).

***Psychotic-like experiences***

PLEs at age 24 years were identified through the face-to-face, semi-structured Psychosis-Like Symptom interview (PLIKSi) (Zammit et al., 2013), designed to assess psychotic experiences in nonclinical populations. It includes 11 core questions about three main positive symptom domains: hallucinations, delusions, and thought interference. Interviews were conducted by trained psychology graduates in assessment clinics. A psychiatrist rated samples of recorded interviews to ensure that ratings were correct. Total scores from the PLIKSi were recoded into a binary variable indicating none versus suspected or definite psychotic experiences that were not attributable to sleep or fever. A full description of the PLIKSi assessment has been described previously (Zammit et al., 2013).

# Statistical analysis

***Colocalization analysis***

LVV-associated CpGs were extracted at a suggestive *p* < 1 × 10^−4^ as the prioritized CpGs for colocalization analyses. We then queried GoDMC (Min et al., 2021) to identify independent cis-mQTLs (*p* < 1 × 10^−8^) associated with the prioritized CpGs. When such mQTLs were identified, we proceeded by extracting all available SNPs associations within a 1 Mb radius from these mQTLs from GoDMC, and from a GWAS of schizophrenia (Lam et al., 2019). We excluded CpG sites with < 10 SNPs available in GoDMC. On the resulting data, a Bayesian colocalization analysis was performed using the *coloc.abf* function with default parameters from the *coloc* R package (Giambartolomei et al., 2018; Wallace, 2020). The method tests five mutually exclusive hypotheses by calculating the posterior probabilities. A posterior probability of a single shared variant (PP_H4_) ≥ 0.8 was considered sufficient evidence for colocalization.

H0: there exist no causal variants for either trait;

H1:there exists a causal variant for trait one only;

H2: there exists a causal variant for trait two only;

H3: there exist two distinct causal variants, one for each trait; and

H4: there exists a single causal variant common to both traits.

**Feature selection using elastic net regularization (ENR)**

The ENR method was used for the informative CpGs feature selection to construct methylation profile scores (MPS). To this end, We split the cord blood DNAm sample into training (80%, *N* = 672) and testing (20%, *N* = 168) datasets using the *createDataPartition* function in the *caret* R package, accounting for even distribution of LVV in the training and testing datasets. The following steps were taken to select the CpG features and estimate coefficients in training set and to evaluate their performance in testing set.

**Step 1. Preselection CpGs.** We preselected LVV-associated CpGs at *p* < 1.0 × 10^−4^ from the cord blood EWAS. The preselected CpGs were used to establish the predictive model in the training set using ENR.

**Step 2. Model building by ENR in the training set.** Optimal combinations of the mixing (alpha) and shrinkage (lambda) parameter were determined via 10-fold cross-validations implemented in the *cva.glmnet* function of *glmnetUtils* package (Friedman et al., 2010). We then extracted the coefficients from the model with the lowest alpha and lambda value corresponding to the minimum mean cross-validated error. The CpGs with zero coefficients were excluded.

**Step 3. The prediction performance testing in the testing set.** CpGs with non-zero coefficients from the elastic net model with the best alpha and lambda values were extracted and used as external weights to construct MPS in cord-blood testing samples. The prediction performance of the ENR-based MPS was evaluated in the testing set to judge direct replication. Specifically, an ENR-weighted DNAm sum score for LVV (i.e. MPS_LVV_) was calculated by multiplying the methylation value at a given CpG by the ENR-based estimated weight, and then summing these values: *MPS_LVV_ = β1*CpG1+β2*CpG2 …+βi*CpGi.* To assess the incremental utility of the MPS_LVV_ over and above covariates, we estimated the incremental R2 by comparing the predictive performance of the full model including the MPS_LVV_ to that of the covariate-only model.

The same approach was repeated based on the cord blood all-male EWAS to construct a male-specific MPS in an independent male sample of ALSPAC _._

**References**

Adriaanse, M., van Domburgh, L., Zwirs, B., Doreleijers, T., & Veling, W. (2015). School-based screening for psychiatric disorders in Moroccan-Dutch youth. *Child and Adolescent Psychiatry and Mental Health, 9*(1), 13.

Bolhuis, K., Koopman-Verhoeff, M. E., Blanken, L. M. E., Cibrev, D., Jaddoe, V. W. V., Verhulst, F. C., et al. (2018). Psychotic-like experiences in pre-adolescence: what precedes the antecedent symptoms of severe mental illness? *Acta Psychiatrica Scandinavica, 138*(1), 15-25.

Boyd, A., Golding, J., Macleod, J., Lawlor, D. A., Fraser, A., Henderson, J., et al. (2013). Cohort Profile: the 'children of the 90s'--the index offspring of the Avon Longitudinal Study of Parents and Children. *Int J Epidemiol, 42*(1), 111-127.

Fischl, B., van der Kouwe, A., Destrieux, C., Halgren, E., Segonne, F., Salat, D. H., et al. (2004). Automatically parcellating the human cerebral cortex. *Cerebral Cortex, 14*(1), 11-22.

Fraser, A., Macdonald-Wallis, C., Tilling, K., Boyd, A., Golding, J., Davey Smith, G., et al. (2013). Cohort Profile: the Avon Longitudinal Study of Parents and Children: ALSPAC mothers cohort. *Int J Epidemiol, 42*(1), 97-110.

Gervin, K., Salas, L. A., Bakulski, K. M., van Zelm, M. C., Koestler, D. C., Wiencke, J. K., et al. (2019). Systematic evaluation and validation of reference and library selection methods for deconvolution of cord blood DNA methylation data. *Clinical Epigenetics, 11*(1).

Harris, P. A., Taylor, R., Thielke, R., Payne, J., Gonzalez, N., & Conde, J. G. (2009). Research electronic data capture (REDCap)—A metadata-driven methodology and workflow process for providing translational research informatics support. *Journal of Biomedical Informatics, 42*(2), 377-381.

Houseman, E. A., Accomando, W. P., Koestler, D. C., Christensen, B. C., Marsit, C. J., Nelson, H. H., et al. (2012). DNA methylation arrays as surrogate measures of cell mixture distribution. *BMC bioinformatics, 13*(1), 1-16.

Ivanova, M. Y., Achenbach, T. M., Rescorla, L. A., Dumenci, L., Almqvist, F., Bilenberg, N., et al. (2007). The generalizability of the Youth Self-Report syndrome structure in 23 societies. *Journal of Consulting and Clinical Psychology, 75*(5), 729-738.

Kaufman, J., Birmaher, B., Brent, D., Rao, U., Flynn, C., Moreci, P., et al. (1997). Schedule for Affective Disorders and Schizophrenia for School-Age Children Present and Lifetime version (K-SADS-PL): Initial reliability and validity data. *Journal of the American Academy of Child and Adolescent Psychiatry, 36*(7), 980-988.

Kooijman, M. N., Kruithof, C. J., van Duijn, C. M., Duijts, L., Franco, O. H., van, I. M. H., et al. (2016). The Generation R Study: design and cohort update 2017. *Eur J Epidemiol, 31*(12), 1243-1264.

Lehne, B., Drong, A. W., Loh, M., Zhang, W., Scott, W. R., Tan, S. T., et al. (2015). A coherent approach for analysis of the Illumina HumanMethylation450 BeadChip improves data quality and performance in epigenome-wide association studies. *Genome Biol, 16*, 37.

Min, J. L., Hemani, G., Davey Smith, G., Relton, C., & Suderman, M. (2018). Meffil: efficient normalization and analysis of very large DNA methylation datasets. *Bioinformatics, 34*(23), 3983-3989.

Relton, C. L., Gaunt, T., McArdle, W., Ho, K., Duggirala, A., Shihab, H., et al. (2015). Data Resource Profile: Accessible Resource for Integrated Epigenomic Studies (ARIES). *Int J Epidemiol, 44*(4), 1181-1190.

Sharp, T. H., McBride, N. S., Howell, A. E., Evans, C. J., Jones, D. K., Perry, G., et al. (2020). Population neuroimaging: generation of a comprehensive data resource within the ALSPAC pregnancy and birth cohort. *Wellcome open research, 5*, 203-203.

White, T., Muetzel, R. L., El Marroun, H., Blanken, L. M. E., Jansen, P., Bolhuis, K., et al. (2018). Paediatric population neuroimaging and the Generation R Study: the second wave. *European Journal of Epidemiology, 33*(1), 99-125.

Zammit, S., Kounali, D., Cannon, M., David, A. S., Gunnell, D., Heron, J., et al. (2013). Psychotic experiences and psychotic disorders at age 18 in relation to psychotic experiences at age 12 in a longitudinal population-based cohort study. *American journal of psychiatry, 170*(7), 742-750.
