# Supplementary material for: DNA methylation at birth and lateral ventricular volume in childhood: a neuroimaging epigenetics study
Source: J Child Psychol Psychiatry. Author manuscript; Available in PMC 2024 Apr 3. (PMC10953396; doi:10.1111/jcpp.13866)
Supplement: Supplementary material [file EMS194680-supplement-Supplementary_material.docx]

**Supplementary Tables and Figures**

**Figure S1. Flow chart of study population selection in Generation R**

Children whose mothers were enrolled in the Generation R Study during pregnancy (*N* = 8,976)

Final EWAS sample for children with available data on cord blood DNAm and LVV at 10 years (*N* = 840)

Children with eligible data on

structural MRI measurement at age10 years (*N* = 3,186)

Children with harmonized cord-blood DNAm data (*N* = 1,382)

Children with eligible data for psychotic-like experience at 14 years

(*N*_hallucinations_ = 4,497;  *N*_delusions_ = 3,659)

Analytic sample for children with available data on MRI and psychotic-like experience

(*N*_hallucinations_ = 2,360, *N*_delusions_= 1,899)

Children with whole blood DNAm data at age10 years

(*N* = 464)

Analytic sample for children with available data on whole blood DNAm and LVV at 10 years (*N* = 370)

**Table S1.** CpG sites in cord blood identified from sex-stratified EWAS as being suggestively associated (*p* < 1×10^−5^) with LVV at age 10 years in Generation R

| **CpG site** | **Chromosome** | **Position** | **Nearest Gene** | **Beta** | **SE** | ***p* value** | **FDR** |
| --- | --- | --- | --- | --- | --- | --- | --- |
| **Male sample (*N* = 417)** | | | | | | | |
| **cg10949007** | **5** | **95159614** | ***GLRX*** | **0.459** | **0.062** | **8.02E-13** | **3.80E-07** |
| **cg22874802** | **1** | **16477614** | ***EPHA2*** | **-0.344** | **0.065** | **2.17E-07** | **0.038** |
| **cg23737062** | **15** | **63894296** | ***FBXL22*** | **-0.371** | **0.070** | **2.43E-07** | **0.038** |
| cg18468519 | 6 | 33944367 | NA | -0.503 | 0.099 | 6.08E-07 | 0.072 |
| cg24440822 | 6 | 27585527 | NA | 0.496 | 0.099 | 8.72E-07 | 0.083 |
| cg03688995 | 11 | 1086421 | *MUC2* | -0.318 | 0.064 | 1.12E-06 | 0.089 |
| cg20995689 | 1 | 243647204 | *SDCCAG8* | -0.360 | 0.074 | 1.88E-06 | 0.116 |
| cg21511395 | 8 | 144942223 | *EPPK1* | -0.382 | 0.080 | 2.41E-06 | 0.116 |
| cg02431851 | 16 | 85543698 | NA | -0.322 | 0.068 | 2.66E-06 | 0.116 |
| cg03748310 | 8 | 145168450 | *KIAA1875* | -0.414 | 0.087 | 2.90E-06 | 0.116 |
| ch.7.1637031F | 7 | 74117341 | *GTF2I* | 0.427 | 0.090 | 3.15E-06 | 0.116 |
| cg16019434 | 18 | 76736785 | NA | 0.350 | 0.074 | 3.52E-06 | 0.116 |
| cg08462497 | 16 | 86411334 | NA | 0.292 | 0.062 | 3.59E-06 | 0.116 |
| cg07245476 | 16 | 60448068 | NA | 0.432 | 0.092 | 3.61E-06 | 0.116 |
| cg00098182 | 15 | 22993172 | *CYFIP1* | -0.322 | 0.068 | 3.66E-06 | 0.116 |
| cg19535267 | 12 | 57625610 | *SHMT2* | -0.321 | 0.070 | 5.93E-06 | 0.170 |
| cg06551661 | 2 | 10691849 | NA | -0.322 | 0.070 | 6.09E-06 | 0.170 |
| cg08170519 | 11 | 133804959 | *IGSF9B* | -0.298 | 0.066 | 7.21E-06 | 0.183 |
| cg07603991 | 17 | 11461103 | *SHISA6* | -0.297 | 0.065 | 7.33E-06 | 0.183 |
| cg15779521 | 12 | 120729960 | NA | 0.393 | 0.087 | 8.64E-06 | 0.195 |
| cg21952528 | 6 | 168721499 | *DACT2* | -0.315 | 0.070 | 8.82E-06 | 0.195 |
| cg19102412 | 2 | 121224327 | *LOC84931* | -0.455 | 0.101 | 9.07E-06 | 0.195 |
| cg15853169 | 2 | 242002555 | *SNED1* | -0.297 | 0.066 | 9.76E-06 | 0.201 |
| **Female sample (*N* = 423)** | | | | | | | |
| cg02299937 | 19 | 40366088 | *FCGBP* | -0.206 | 0.039 | 2.91E-07 | 0.138 |
| cg18950108 | 6 | 30920171 | *DPCR1* | -0.248 | 0.051 | 1.52E-06 | 0.361 |
| cg08557523 | 2 | 220379968 | *ACCN4* | -0.247 | 0.052 | 3.22E-06 | 0.503 |
| cg17532978 | 12 | 55413394 | *NEUROD4* | -0.196 | 0.042 | 4.93E-06 | 0.503 |
| cg17703554 | 8 | 88886339 | *DCAF4L2* | -0.201 | 0.043 | 5.31E-06 | 0.503 |
| cg00741731 | 19 | 42348676 | *LYPD4;DMRTC2* | -0.305 | 0.068 | 9.41E-06 | 0.612 |
| cg27599319 | 17 | 18680666 | *FBXW10* | 0.258 | 0.058 | 9.73E-06 | 0.612 |

Note. NA= not available. The full model is adjusted for batch effects, estimated cell-type proportions, gestational age, maternal age in take/delivery, maternal smoking during pregnancy, child age at MRI assessment and total brain volume

**Figure S2.** Manhattan plots for sex-stratified EWAS between DNAm at birth and LVV at age 10 years in Generation R


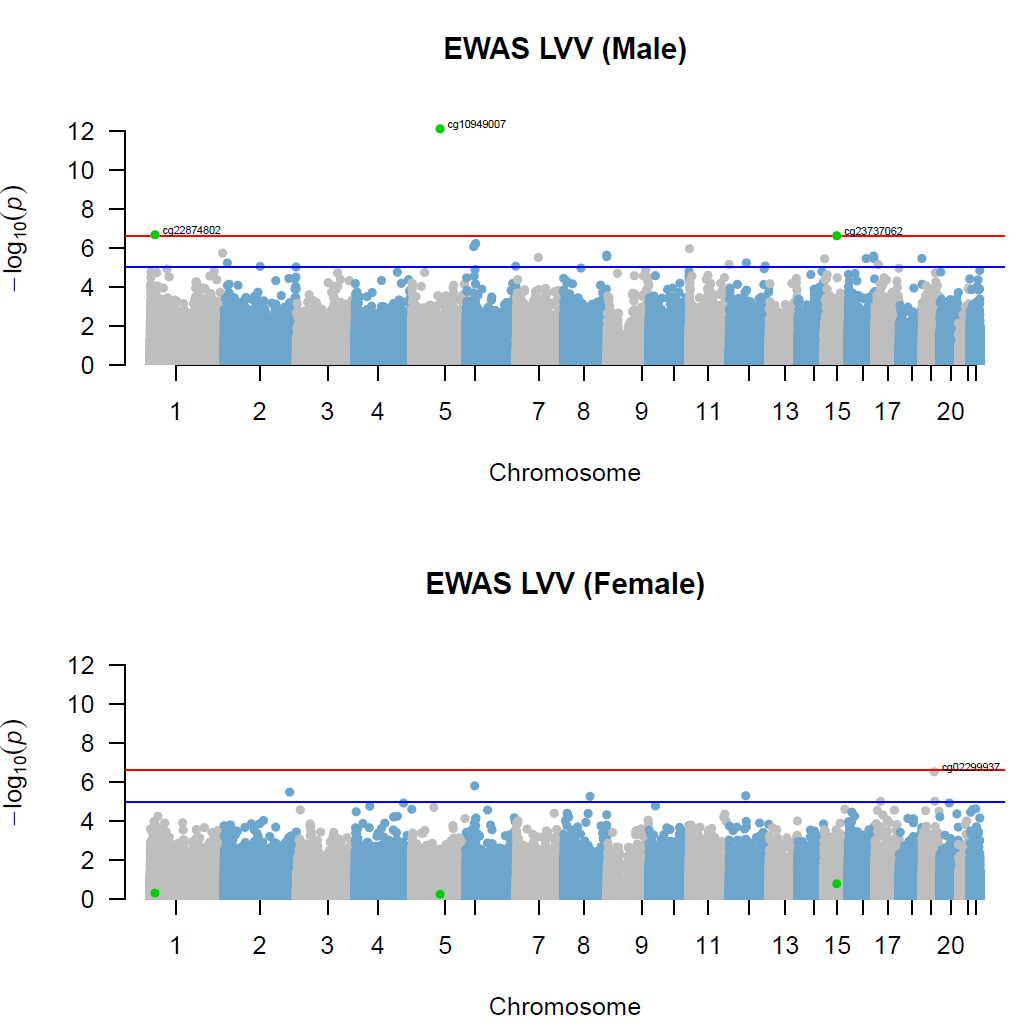


**Table S2.** Correlation between blood and brain methylation based on two independent online comparison tools, at top LVV-associated CpGs from the cord blood EWAS

| **CpG site** | **Chromosome** | **Position** | **Genes** | **Image-CpG (*N* = 13)** |  | **DNA Methylation Comparison Tool (*N* = 71-75)** | | | |
| --- | --- | --- | --- | --- | --- | --- | --- | --- | --- |
|  |  |  |  | **Gray matter** |  | **PFC** | **EC** | **STG** | **CER** |
| cg23923495 | 1 | 215741843 | *KCTD3* | NA |  | 0.23 | 0.08 | 0.17 | -0.16 |
| cg20995689 | 1 | 243647204 | *SDCCAG8* | -0.40 |  | -0.10 | -0.01 | -0.15 | -0.02 |
| cg08945340 | 16 | 1077028 | NA | 0.62 |  | 0.20 | 0.08 | 0.18 | 0.06 |
| cg10949007 | 5 | 95159614 | *GLRX* | -0.30 |  | -0.08 | 0.00 | -0.10 | 0.16 |

Note. Image-CpG at <https://han-lab.org/methylation/default/imageCpG>; Blood Brain DNA Methylation Comparison Tool at <https://epigenetics.essex.ac.uk/bloodbrain/?probenameg>; PFC= prefrontal cortex, EC= entorhinal cortex, STG= superior temporal gyrus, CER=cerebellum

**Figure S3a.** Heatmap depicting the average expression of genes annotated to the top LVV-associated CpGs across 54 tissues provided by GTEx.


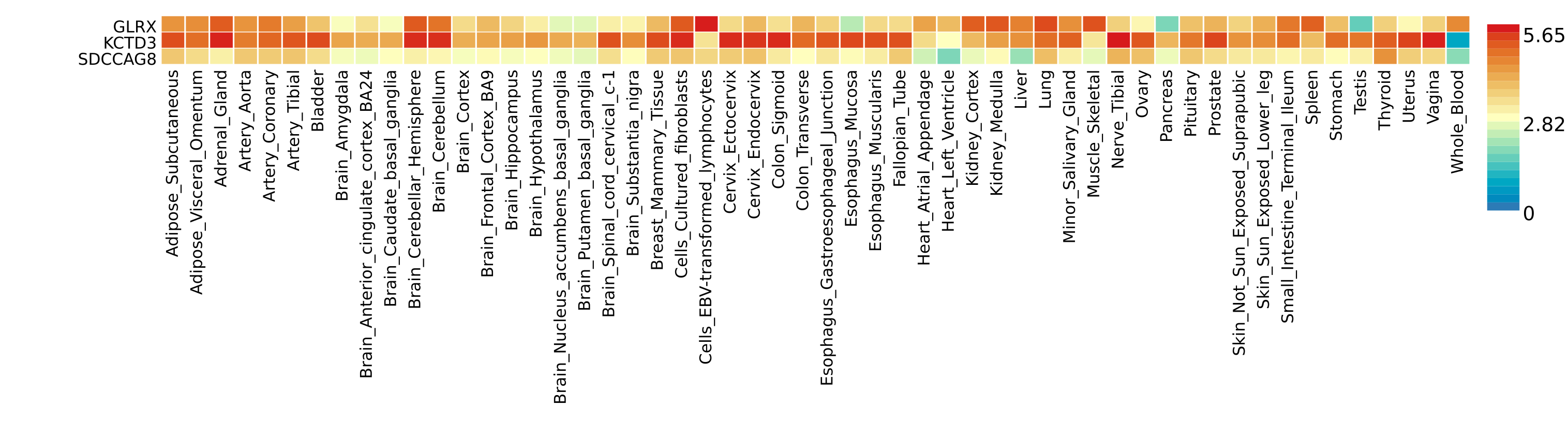


Note. Colors indicate the average expression value (log2 transformed Reads Per Kilobase per Million per tissue per gene). Darker red color indicates higher expression of the gene, while darker blue represents lower expression level.

**Figure S3b.** Heatmap depicting the average expression of genes annotated to the LVV-associated CpGs across 11 general developmental stages across brain samples provided by BrainSpan.


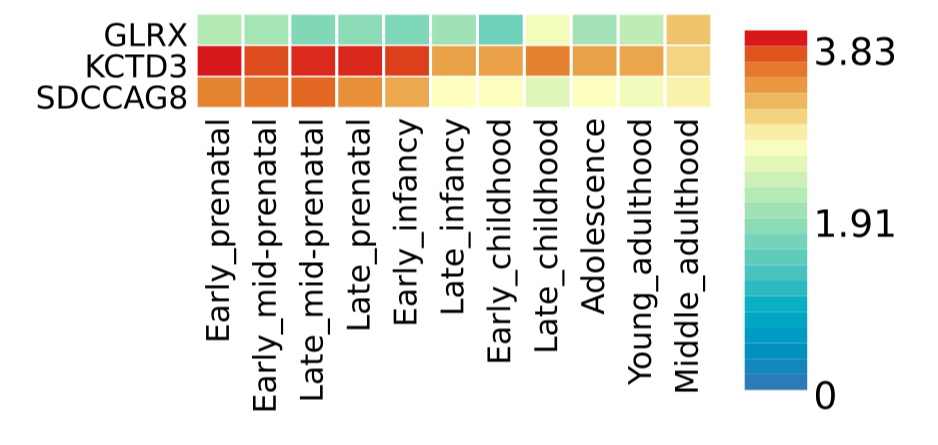


Note. Colors indicate the average expression value. Darker red color indicates higher expression of the gene, while darker blue represents lower expression level. This figure was downloaded from the official BrainSpan atlas through FUMA GWAS (<https://fuma.ctglab.nl/>).

**Table S3.** Significant mQTLs from GoDMC (<http://mqtldb.godmc.org.uk/>) for two top CpGs from the cord blood EWAS

| **mQTL** | **A1** | **A2** | **CpG site** | **Beta** | **SE** | **Sample size** | ***p* value** |
| --- | --- | --- | --- | --- | --- | --- | --- |
| rs9314160 | A | G | cg10949007 | -0.120 | 0.009 | 26646 | 5.76E-43 |
| rs3756705 | T | G | cg10949007 | 0.125 | 0.009 | 27255 | 1.55E-42 |
| rs7700327 | C | T | cg10949007 | 0.125 | 0.009 | 27243 | 1.83E-42 |
| rs6556884 | A | G | cg10949007 | 0.123 | 0.010 | 27255 | 2.39E-35 |
| rs6556885 | A | G | cg10949007 | 0.121 | 0.010 | 27254 | 1.64E-34 |
| rs6875489 | C | T | cg10949007 | -0.109 | 0.009 | 26621 | 9.72E-33 |
| rs3756704 | A | G | cg10949007 | 0.090 | 0.009 | 26438 | 2.49E-25 |
| rs4561 | G | A | cg10949007 | -0.090 | 0.009 | 26649 | 7.53E-25 |
| rs1047420 | A | G | cg10949007 | -0.090 | 0.009 | 27141 | 9.93E-25 |
| rs28926210 | A | G | cg10949007 | -0.091 | 0.009 | 26588 | 2.48E-24 |
| rs62013848 | T | C | cg08945340 | 0.410 | 0.019 | 24588 | 1.97E-107 |
| rs62013851 | A | C | cg08945340 | 0.387 | 0.018 | 24103 | 5.30E-99 |
| rs2573148 | T | C | cg08945340 | 0.245 | 0.012 | 25864 | 1.93E-94 |
| rs2744148 | G | A | cg08945340 | 0.244 | 0.012 | 25885 | 3.76E-94 |
| rs400354 | G | T | cg08945340 | 0.242 | 0.012 | 25843 | 1.65E-92 |
| rs449295 | A | G | cg08945340 | 0.242 | 0.012 | 25849 | 1.91E-92 |
| rs62013844 | T | C | cg08945340 | 0.397 | 0.020 | 21839 | 1.00E-90 |
| rs9936536 | A | G | cg08945340 | 0.344 | 0.017 | 24590 | 2.24E-90 |
| rs449566 | T | C | cg08945340 | 0.242 | 0.012 | 25098 | 1.10E-89 |
| rs75697062 | T | C | cg08945340 | 0.395 | 0.020 | 21833 | 2.52E-89 |

Note. A total number of 22 and 56 cis-mQTL associations were identified at p< 1×10^-8^ for cg10949007 and cg08945340, respectively. Here we list the top 10 significant mQTLs sorted by *p* value.

**Table S4.** Colocalization of independent genetic variants across schizophrenia (SCZ) and mQTL, PP = posterior probability, sorted by PPH4

| **Trait 1** | **Trait 2** | **Index mQTL** | **CpG site** | **Chr:Pos (Index mQTL)** | **N of SNPs** | **PP.H4.abf** |
| --- | --- | --- | --- | --- | --- | --- |
| **DNAm** | **SCZ** | **rs3802923** | **cg08170519** | **11:133786824** | **30** | **0.981** |
| DNAm | SCZ | rs4843687 | cg06928952 | 16:87762057 | 291 | 0.351 |
| DNAm | SCZ | rs41345548 | cg03688995 | 11:1080223 | 720 | 0.343 |
| DNAm | SCZ | rs4552148 | cg08806184 | 2:27080513 | 510 | 0.174 |
| DNAm | SCZ | rs935734 | cg07438401 | 3:67699371 | 288 | 0.104 |
| DNAm | SCZ | rs2240803 | cg18950108 | 6:30920957 | 30 | 0.048 |
| DNAm | SCZ | rs2162480 | cg01371799 | 2:231130887 | 169 | 0.042 |
| DNAm | SCZ | rs56317957 | cg01371799 | 2:231162955 | 169 | 0.042 |
| DNAm | SCZ | rs71608429 | cg05234599 | 4:142119340 | 74 | 0.034 |
| DNAm | SCZ | rs138997233 | cg22874802 | 1:16351872 | 48 | 0.034 |
| DNAm | SCZ | rs7415766 | cg01960096 | 1:156947358 | 381 | 0.026 |
| DNAm | SCZ | rs79524843 | cg14697334 | 17:37832386 | 98 | 0.023 |
| DNAm | SCZ | rs2882498 | cg18204120 | 14:60684321 | 692 | 0.021 |
| DNAm | SCZ | rs17805820 | cg18204120 | 14:61652619 | 693 | 0.021 |
| DNAm | SCZ | rs62187472 | cg21493483 | 2:236044045 | 38 | 0.019 |
| DNAm | SCZ | rs141962785 | cg09737078 | 16:4309369 | 230 | 0.016 |
| DNAm | SCZ | rs73271930 | cg21615831 | 17:25822224 | 19 | 0.016 |
| DNAm | SCZ | rs77105837 | cg09158990 | 12:49277680 | 54 | 0.014 |
| DNAm | SCZ | rs6441602 | cg02570223 | 3:101123041 | 104 | 0.014 |
| DNAm | SCZ | rs12220398 | cg00320980 | 10:134748531 | 588 | 0.014 |
| DNAm | SCZ | rs7093215 | cg00320980 | 10:134813796 | 588 | 0.014 |
| DNAm | SCZ | rs9314160 | cg10949007 | 5:95156457 | 22 | 0.013 |
| DNAm | SCZ | rs4709012 | cg05495478 | 6:164173156 | 418 | 0.013 |
| DNAm | SCZ | rs4709741 | cg05495478 | 6:164092291 | 418 | 0.013 |
| DNAm | SCZ | rs12600016 | cg08945340 | 16:1081175 | 58 | 0.013 |
| DNAm | SCZ | rs3815332 | cg03348584 | 7:151092775 | 39 | 0.013 |
| DNAm | SCZ | rs7768886 | cg21952528 | 6:168728989 | 28 | 0.012 |
| DNAm | SCZ | rs11102515 | cg03884543 | 1:109371874 | 317 | 0.012 |
| DNAm | SCZ | rs2787088 | cg08284598 | 20:3645916 | 833 | 0.010 |
| DNAm | SCZ | rs41281858 | cg08284598 | 20:3209083 | 833 | 0.010 |
| DNAm | SCZ | rs540269 | cg15526825 | 11:75151168 | 246 | 0.010 |
| DNAm | SCZ | rs11236457 | cg15526825 | 11:75274061 | 248 | 0.010 |
| DNAm | SCZ | rs715930 | cg07962360 | 12:58023981 | 236 | 0.009 |
| DNAm | SCZ | rs11722394 | cg18636319 | 4:26456376 | 219 | 0.009 |
| DNAm | SCZ | rs351890 | cg02197387 | 20:62804629 | 763 | 0.009 |
| DNAm | SCZ | rs181239016 | cg18269143 | 22:32336796 | 74 | 0.009 |
| DNAm | SCZ | rs7761922 | cg01128736 | 6:4456369 | 12 | 0.008 |
| DNAm | SCZ | rs350217 | cg02535219 | 16:12150474 | 327 | 0.008 |
| DNAm | SCZ | rs8045673 | cg02535219 | 16:11804385 | 327 | 0.008 |
| DNAm | SCZ | rs3929570 | cg01899620 | 19:37556081 | 10 | 0.008 |
| DNAm | SCZ | rs1667342 | cg02570223 | 19:37468696 | 556 | 0.008 |
| DNAm | SCZ | rs2672779 | cg14722290 | 5:435055 | 44 | 0.008 |
| DNAm | SCZ | rs2359013 | cg02902761 | 4:7027828 | 71 | 0.008 |
| DNAm | SCZ | rs10800458 | cg16054275 | 1:169549811 | 162 | 0.008 |
| DNAm | SCZ | rs3917731 | cg16054275 | 1:169580717 | 162 | 0.008 |
| DNAm | SCZ | rs748206 | cg15935315 | 6:5949593 | 142 | 0.008 |
| DNAm | SCZ | rs67726376 | cg11369761 | 2:168032190 | 46 | 0.008 |
| DNAm | SCZ | rs12242379 | cg03109992 | 10:134741143 | 58 | 0.007 |
| DNAm | SCZ | rs7069085 | cg03109992 | 10:134885677 | 58 | 0.007 |
| DNAm | SCZ | rs3794702 | cg06723414 | 16:3730613 | 384 | 0.007 |
| DNAm | SCZ | rs116335392 | cg08930131 | 5:177961140 | 474 | 0.007 |
| DNAm | SCZ | rs7706759 | cg08930131 | 5:177893248 | 475 | 0.007 |
| DNAm | SCZ | rs113599906 | cg00202760 | 16:88875386 | 280 | 0.007 |
| DNAm | SCZ | rs837763 | cg00202760 | 16:88853729 | 280 | 0.007 |
| DNAm | SCZ | rs61089964 | cg07245476 | 16:60447948 | 18 | 0.007 |
| DNAm | SCZ | rs62033202 | cg26806527 | 16:17387241 | 25 | 0.007 |
| DNAm | SCZ | rs2641772 | cg20710258 | 3:195531841 | 107 | 0.007 |
| DNAm | SCZ | rs62049562 | cg27072191 | 16:77211020 | 772 | 0.007 |
| DNAm | SCZ | rs9928113 | cg27072191 | 16:76908524 | 772 | 0.007 |
| DNAm | SCZ | rs12371717 | cg16379671 | 12:131912479 | 516 | 0.006 |
| DNAm | SCZ | rs4759536 | cg16379671 | 12:131495766 | 516 | 0.006 |
| DNAm | SCZ | rs7487651 | cg16379671 | 12:131636966 | 516 | 0.006 |
| DNAm | SCZ | rs1819424 | cg23821359 | 13:112862643 | 565 | 0.006 |
| DNAm | SCZ | rs2993313 | cg01899620 | 13:113684467 | 207 | 0.006 |
| DNAm | SCZ | rs3011530 | cg01899620 | 13:113648592 | 207 | 0.006 |
| DNAm | SCZ | rs17765097 | cg08140055 | 3:134816417 | 914 | 0.006 |
| DNAm | SCZ | rs13067564 | cg08140055 | 3:134209956 | 923 | 0.006 |
| DNAm | SCZ | rs71331775 | cg08140055 | 3:134462867 | 923 | 0.006 |
| DNAm | SCZ | rs16970288 | cg24750752 | 19:35887129 | 50 | 0.006 |
| DNAm | SCZ | rs1860339 | cg00648184 | 17:60851264 | 106 | 0.006 |
| DNAm | SCZ | rs5751614 | cg25410636 | 22:23593051 | 16 | 0.006 |
| DNAm | SCZ | rs10744006 | cg26244225 | 12:12931017 | 76 | 0.006 |
| DNAm | SCZ | rs34323 | cg26244225 | 12:12879057 | 76 | 0.006 |
| DNAm | SCZ | rs7925722 | cg10376408 | 11:9120068 | 278 | 0.006 |
| DNAm | SCZ | rs9640266 | cg00289558 | 7:149566175 | 103 | 0.006 |
| DNAm | SCZ | rs2016598 | cg21845080 | 19:38141758 | 120 | 0.005 |
| DNAm | SCZ | rs7739936 | cg05130022 | 6:26575443 | 107 | 0.000 |

**Table S5a.** Top 20 GO terms for LVV-associated CpGs at a threshold of *p* < 1 × 10^−4^

|  | **Ontology** | | **Term** | **N Genes in Term** | **N Differentially Methylated Genes** | ***p* value** | **FDR** | |
| --- | --- | --- | --- | --- | --- | --- | --- | --- |
| GO:0045924 | | BP | regulation of female receptivity | 8 | 2 | 0.0007 | | 1 |
| GO:0060180 | | BP | female mating behavior | 8 | 2 | 0.0007 | | 1 |
| GO:0007162 | | BP | negative regulation of cell adhesion | 286 | 7 | 0.0014 | | 1 |
| GO:0080058 | | BP | protein deglutathionylation | 1 | 1 | 0.0019 | | 1 |
| GO:0098754 | | BP | detoxification | 143 | 4 | 0.0022 | | 1 |
| GO:1990961 | | BP | xenobiotic detoxification by transmembrane export across the plasma membrane | 18 | 2 | 0.0023 | | 1 |
| GO:0031595 | | CC | nuclear proteasome complex | 1 | 1 | 0.0025 | | 1 |
| GO:0022408 | | BP | negative regulation of cell-cell adhesion | 185 | 5 | 0.0028 | | 1 |
| GO:0004757 | | MF | sepiapterin reductase activity | 1 | 1 | 0.0030 | | 1 |
| GO:0061830 | | CC | concave side of sperm head | 1 | 1 | 0.0031 | | 1 |
| GO:0072055 | | BP | renal cortex development | 1 | 1 | 0.0032 | | 1 |
| GO:0072059 | | BP | cortical collecting duct development | 1 | 1 | 0.0032 | | 1 |
| GO:0072214 | | BP | metanephric cortex development | 1 | 1 | 0.0032 | | 1 |
| GO:0072219 | | BP | metanephric cortical collecting duct development | 1 | 1 | 0.0032 | | 1 |
| GO:0030020 | | MF | extracellular matrix structural constituent conferring tensile strength | 39 | 3 | 0.0033 | | 1 |
| GO:0046618 | | BP | xenobiotic export | 20 | 2 | 0.0034 | | 1 |
| GO:0045244 | | CC | succinate-CoA ligase complex (GDP-forming) | 1 | 1 | 0.0038 | | 1 |
| GO:0061775 | | MF | cohesin loading activity | 1 | 1 | 0.0038 | | 1 |
| GO:0004362 | | MF | glutathione-disulfide reductase (NADPH) activity | 2 | 1 | 0.0039 | | 1 |
| GO:0045623 | | BP | negative regulation of T-helper cell differentiation | 17 | 2 | 0.0039 | | 1 |

**Table S5b.** Top 20 KEGG pathways for LVV-associated CpGs at a threshold of *p* < 1 × 10^−4^

|  | **Term** | ***N* Genes in Term** | | | ***N* Differentially Methylated Genes** | | ***p* value** | **FDR** | | |
| --- | --- | --- | --- | --- | --- | --- | --- | --- | --- | --- |
| path:hsa04974 | Protein digestion and absorption | | 92 | 3 | | 0.0184 | | | 1 |  |
| path:hsa00120 | Primary bile acid biosynthesis | | 17 | 1 | | 0.0539 | | | 1 |  |
| path:hsa04662 | B cell receptor signaling pathway | | 78 | 2 | | 0.0674 | | | 1 |  |
| path:hsa05022 | Pathways of neurodegeneration - multiple diseases | | 444 | 5 | | 0.0823 | | | 1 |  |
| path:hsa00790 | Folate biosynthesis | | 26 | 1 | | 0.0907 | | | 1 |  |
| path:hsa00020 | Citrate cycle (TCA cycle) | | 28 | 1 | | 0.0972 | | | 1 |  |
| path:hsa04934 | Cushing syndrome | | 154 | 3 | | 0.1007 | | | 1 |  |
| path:hsa00604 | Glycosphingolipid biosynthesis - ganglio series | | 15 | 1 | | 0.1045 | | | 1 |  |
| path:hsa04390 | Hippo signaling pathway | | 154 | 3 | | 0.1097 | | | 1 |  |
| path:hsa04512 | ECM-receptor interaction | | 86 | 2 | | 0.1148 | | | 1 |  |
| path:hsa04670 | Leukocyte transendothelial migration | | 106 | 2 | | 0.1196 | | | 1 |  |
| path:hsa00534 | Glycosaminoglycan biosynthesis - heparan sulfate / heparin | | 23 | 1 | | 0.1321 | | | 1 |  |
| path:hsa00532 | Glycosaminoglycan biosynthesis - chondroitin sulfate / dermatan sulfate | | 20 | 1 | | 0.1366 | | | 1 |  |
| path:hsa00640 | Propanoate metabolism | | 32 | 1 | | 0.1435 | | | 1 |  |
| path:hsa04510 | Focal adhesion | | 195 | 3 | | 0.1530 | | | 1 |  |
| path:hsa05165 | Human papillomavirus infection | | 313 | 4 | | 0.1559 | | | 1 |  |
| path:hsa03050 | Proteasome | | 45 | 1 | | 0.1578 | | | 1 |  |
| path:hsa00140 | Steroid hormone biosynthesis | | 57 | 1 | | 0.1691 | | | 1 |  |
| path:hsa04014 | Ras signaling pathway | | 226 | 3 | | 0.1745 | | | 1 |  |
| path:hsa04110 | Cell cycle | | 152 | 2 | | 0.1782 | | | 1 |  |

**Table S6a.** Pearson's correlation coefficient of DNAm level between two time points (at birth vs. childhood) in Generation R (*N* = 448), at four top CpGs from the cord blood EWAS

| **CpG site** | **Chromosome** | **Position** | **Nearest Gene** | ***r*** | ***p* value** |
| --- | --- | --- | --- | --- | --- |
| cg23923495 | 1 | 215741843 | *KCTD3* | 0.093 | 0.049 |
| cg20995689 | 1 | 243647204 | *SDCCAG8* | -0.035 | 0.462 |
| cg08945340 | 16 | 1077028 | NA | 0.180 | **0.0001** |
| cg10949007 | 5 | 95159614 | *GLRX* | 0.055 | 0.239 |

**Table S6b.** Comparison between prospective and cross-sectional associations in Generation R, at four top CpGs from the cord blood EWAS

| **CpG site** | **Chromosome** | **Position** | **Nearest**  **Gene** | **Prospective association (*N* = 840)** | | |  | **Cross-sectional association (*N* = 370)** | | |  | **Direction** |
| --- | --- | --- | --- | --- | --- | --- | --- | --- | --- | --- | --- | --- |
|  |  |  |  | **Beta** | **SE** | ***p* value** |  | **Beta** | **SE** | ***p* value** |  |  |
| cg23923495 | 1 | 215741843 | *KCTD3* | -0.259 | 0.046 | 2.08E-08 |  | 0.023 | 0.063 | 0.722 |  | -+ |
| cg20995689 | 1 | 243647204 | *SDCCAG8* | -0.252 | 0.046 | 7.51E-08 |  | 0.042 | 0.056 | 0.452 |  | -+ |
| cg08945340 | 16 | 1077028 | NA | -0.218 | 0.041 | 1.37E-07 |  | -0.037 | 0.059 | 0.529 |  | -- |
| cg10949007 | 5 | 95159614 | *GLRX* | 0.205 | 0.039 | 1.68E-07 |  | 0.060 | 0.060 | 0.318 |  | ++ |

**Table S7a.**  Regression results for top four CpGs identified from sex-combined EWAS in the overall sample of Generation R and the probe-level replication in ALSPAC, stratified by child sex

| **CpG site** | **Chr** | **Position** | **Nearest Gene** | **Generation R_Female**  **(*N* = 423)** | | |  | **Generation R_Male**  **(*N* = 417)** | | |  | **ALSPAC_Male**  **(*N* = 114)** | | | **Direction** |
| --- | --- | --- | --- | --- | --- | --- | --- | --- | --- | --- | --- | --- | --- | --- | --- |
|  |  |  |  | **Beta** | **SE** | ***p* value** |  | **Beta** | **SE** | ***p* value** |  | **Beta** | **SE** | ***p* value** |  |
| cg23923495 | 1 | 215741843 | *KCTD3* | -0.21 | 0.06 | 0.002 |  | -0.29 | 0.07 | 1.62E-05 |  | -0.01 | 0.09 | 0.93 | --- |
| cg20995689 | 1 | 243647204 | *SDCCAG8* | -0.19 | 0.07 | 0.011 |  | -0.32 | 0.06 | 1.88E-06 |  | -0.07 | 0.11 | 0.51 | --- |
| cg08945340 | 16 | 1077028 | NA | -0.14 | 0.06 | 0.018 |  | -0.26 | 0.06 | 2.31E-05 |  | 0.11 | 0.12 | 0.38 | --+ |
| cg10949007 | 5 | 95159614 | *GLRX* | -0.03 | 0.06 | 0.556 |  | 0.40 | 0.05 | **8.02E-13** |  | 0.13 | 0.12 | 0.28 | -++ |

**Table S7b.** Regression results for top three CpGs identified from the all-male EWAS in Generation R and the probe-level replication in ALSPAC.

| **CpG site** | **Chr** | **Position** | **Nearest Gene** | **Generation R_Male**  **(*N* = 417)** | | |  | **ALSPAC_Male**  **(*N* = 114)** | | | **Direction** |
| --- | --- | --- | --- | --- | --- | --- | --- | --- | --- | --- | --- |
|  |  |  |  | **Beta** | **SE** | ***p* value** |  | **Beta** | **SE** | ***p* value** |  |
| cg10949007 | 5 | 95159614 | *GLRX* | 0.40 | 0.05 | 8.02E-13 |  | 0.13 | 0.12 | 0.28 | ++ |
| cg22874802 | 1 | 16477614 | *EPHA2* | -0.34 | 0.07 | 2.17E-07 |  | -0.06 | 0.11 | 0.59 | -- |
| cg23737062 | 15 | 63894296 | *FBXL22* | -0.37 | 0.07 | 2.44E-07 |  | 0.08 | 0.12 | 0.51 | -+ |
